# Integrated GC-MS Fingerprinting and Chemometrics for Identifying Antimicrobial Quality Markers in *Bidens pilosa* L. Volatile Oils

**DOI:** 10.3390/plants15172665

**Published:** 2026-08-31

**Authors:** Yuhang Jiang, Qingyu Cheng, Yebing Zhang, Xiaoqin Lin, Longxin Qiu

**Affiliations:** 1 College of Life Science, Longyan University, Longyan 364000, China; 15687083331@163.com (Q.C.); 15280800904@163.com (Y.Z.); qlongxin@tom.com (L.Q.); 2 School of Resource Engineering, Longyan University, Longyan 364000, China; lxqfj90@163.com

**Keywords:** *Bidens pilosa*, volatile oil, antimicrobial activity, phytochemical profiling, quality marker

## Abstract

*Bidens pilosa* L. is an herbal medicine rich in volatile oils (VOs) with anti-inflammatory, antioxidant, and antimicrobial activities. However, the volatile constituents responsible for its antimicrobial effect (quality-indicators, Q-markers) have not been clearly defined, limiting product standardization. In this study, VOs from eleven sites in China were obtained by supercritical-fluid extraction and analyzed by GC-MS, yielding 75 compounds and a fingerprint with a similarity > 0.90 based on 43 common peaks. Chemometric screening using relative abundance, followed by hierarchical cluster analysis (HCA) and principal component analysis (PCA) with variable-importance-in-projection (VIP) scores, divided the oils into three geographical clusters and identified 14 differential compounds (VIP ≥ 1, *p* < 0.05). Antimicrobial assays against *E. coli*, *S. aureus*, *S. enterica* and *P. multocida* revealed pronounced regional variation, with Guangdong, Fujian and Jiangxi extracts showing broad activity. Spectrum–effect correlation analysis identified methyl laurate (0.832–0.976), (Z)-3-heptadecen-5-yne (0.757–0.997), stearic acid (0.815–0.919) and stearolic acid (0.888–0.960) as candidate quality markers, based on their statistical association with antibacterial activity. Mechanistic studies and validation of these candidate markers are necessary to confirm their individual and synergistic roles and to establish a definitive standardized quality control method.

## 1. Introduction

*Bidens pilosa* L. (*Asteraceae*), a widely distributed annual herb, is recognized in traditional medicine globally for its diverse therapeutic properties, including anti-inflammatory, antioxidant, and antimicrobial activities [1,2]. Its aerial parts have been empirically used for centuries to manage a wide array of ailments, including inflammatory conditions, microbial infections, gastrointestinal disorders, and hypertension [1,2,3,4]. Its volatile oils (VOs), representing a significant fraction of its bioactive constituents, have demonstrated efficacy against clinically relevant pathogens such as *Escherichia coli* and *Staphylococcus aureus* [5,6,7]. The increasing global demand for natural antimicrobial agents in human and animal health necessitates reliable quality control and therapeutic standardization of such botanical resources [8].

However, the phytochemical composition and consequently the bioactivity of *B. pilosa* VOs exhibit significant variability due to the species’ ecological adaptability to diverse geographical environments [9]. This chemotypic heterogeneity poses a substantial challenge for the consistent application of *B. pilosa* VOs as standardized therapeutic agents. While previous studies have identified various volatile compounds in *B. pilosa* [10,11] and explored its general antimicrobial effects [7,12], these investigations have largely remained at the level of compositional description or broad pharmacological observation. In particular, they have not systematically integrated volatile oil fingerprinting with antibacterial validation across multiple geographical origins, nor have they identified a set of bioactivity-associated chemical markers that could support reproducible quality assessment and practical standardization. This deficiency hinders the development of robust quality control frameworks and limits the reliable translation of laboratory findings into pharmaceutical applications [13]. Gas chromatography–mass spectrometry (GC-MS) coupled with chemometric analysis has emerged as a powerful technique for establishing chemical fingerprints and discriminating between geographical chemotypes of medicinal plants [14,15,16]. This approach, when integrated with bioactivity evaluation, is instrumental in identifying Q-markers [17,18].

The Q-marker concept, originally proposed in the context of Chinese Materia Medica (CMM), provides a particularly suitable framework for addressing this gap because it emphasizes not only the detectability and specificity of chemical constituents, but also their relevance to biological efficacy and quality consistency. For volatile botanical products such as *B. pilosa* oils, whose chemical profiles are strongly influenced by habitat and origin, the Q-marker strategy is uniquely valuable in moving beyond conventional “component identification” toward efficacy-oriented quality control. By linking chromatographic signals with antibacterial performance, this concept enables the screening of compounds that are both analytically traceable and pharmacologically meaningful, thereby offering a more rational basis for standardization than compositional profiling alone. Despite the success of these methodologies in other botanical species, a standardized GC-MS fingerprint, rigorously validated by bioactivity and linked to specific Q-markers for *B. pilosa* volatile oils, remains critically unestablished. Accordingly, the novelty of the present study lies not merely in profiling the volatile constituents of *B. pilosa*, but in integrating geographical chemotype discrimination, antibacterial activity evaluation, and spectrum–effect relationship analysis to identify bioactivity-anchored Q-markers within a unified quality assessment framework. Therefore, this study aims to address these critical limitations by: (1) developing a robust and reproducible GC-MS fingerprint for *B. pilosa* volatile oils; (2) utilizing chemometric analysis (hierarchical cluster analysis and principal component analysis) to discriminate biogeographical chemotypes and identify geographically relevant chemical variations; (3) conducting comprehensive in vitro antibacterial efficacy evaluations against key priority pathogens, including *Escherichia coli*, *Staphylococcus aureus*, *Salmonella enterica*, and *Pasteurella multocida*, which represent critical threats in public health and veterinary medicine; and (4) identifying bioactivity-anchored quality markers (Q-markers) through spectrum‒effect modeling (Gray Relational Analysis). This integrated, tripartite methodology, connecting chemical profiling with biological validation, will provide a crucial quality control framework, enabling the reproducible development and reliable application of *B. pilosa*-derived antimicrobial agents in pharmaceutical development and as evidence-based natural therapeutics.

## 2. Results

### 2.1. Yield and Chemical Composition of Volatile Oils from B. pilosa from Different Production Areas

To investigate the variability in volatile oil production and composition across different geographical sources, eleven batches of *B. pilosa* were collected from eleven distinct regions and subjected to volatile oil extraction. The experimental results revealed significant regional variation in volatile oil yields, with samples from S7, S9, and S11 demonstrating relatively high yields, exceeding 2% (Table 1). In contrast, samples from Shijiazhuang (Hebei Province), Nanyang (Henan Province), and Guiyang (Guizhou Province) presented lower volatile oil yields. These findings highlight the substantial regional differences in the volatile oil content of different *B. pilosa* sources, suggesting the influence of geographical and environmental factors on the biosynthesis and accumulation of these compounds. Notably, samples from Jiangxi Province exhibited signs of varietal confusion and inconsistent quality, pointing to variability, potentially in either genetic or environmental factors.

Subsequently, the chemical constituents of these extracted volatile oils were analyzed using Gas Chromatography–Mass Spectrometry (GC‒MS). Compound annotation was based on harmonized criteria combining NIST 2014 spectral similarity (≥90%) and RI agreement (ΔRI ≤ ±10). Based on the retention indices and spectral matches, a total of 75 compounds were identified across all samples. These identified compounds included 13 esters, 13 sesquiterpenes, 9 alkanes, 6 monoterpenes, 6 phytosterols, 5 alcohols, 5 fatty acids, 4 triterpenes, 3 aldehydes, 2 ketones, 2 alkynes, 1 diterpene, 1 alkaloid, 1 phenol, and 4 other compounds (Table S3). The relative contents of these constituents were quantified via the normalized peak area method. The overall chemical profile indicated that the volatile oils predominantly comprised terpenoids and their derivatives, such as esters, ketones, alkanes, phytosterols, alkynes, and alkaloids. Prominent monoterpenes identified included cis-nerolidol, manool, and iso-longifolene, while prevalent sesquiterpenes encompassed caryophyllene, α-humulene, and β-elemene oxide. Other significant compounds identified across samples included methyl laurate (with a relative abundance exceeding 25%), γ- muurolene (greater than 20%), stearic acid (exceeding 10%), and stearolic acid (surpassing 8%). The dominance of terpenoids, particularly monoterpenes and sesquiterpenes, across different geographic origins of *B. pilosa* reflects species-specific characteristics and observed environmental influences on the chemical composition of the essential oils. These variations in chemical composition are likely to have implications for the pharmacological activities of the volatile oils from different regions.

### 2.2. Fingerprint Analysis and Similarity Assessment of Volatile Oils from B. pilosa

**Figure 1:**
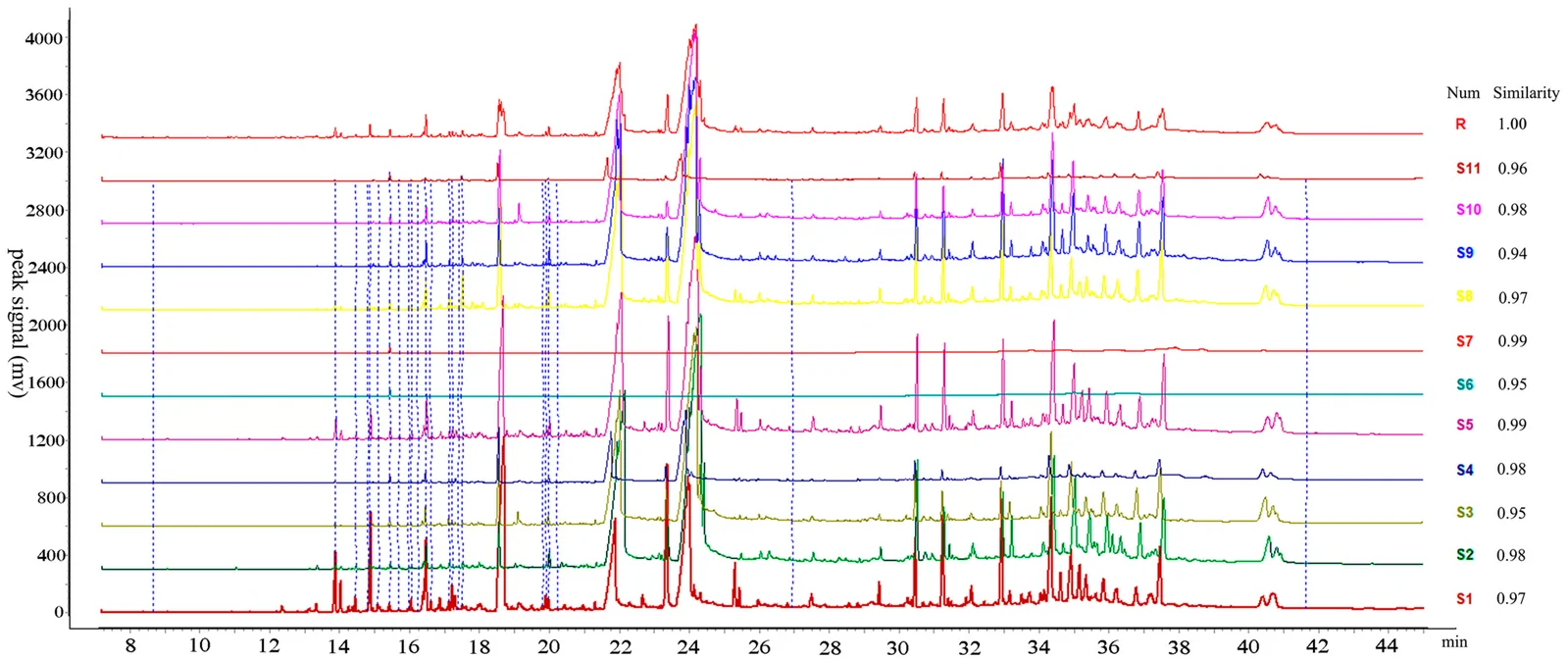
Gas Chromatography–Mass Spectrometry (GC-MS) fingerprints of volatile oils from 11 batches of *B.s pilosa* L. Note: S1_Shijiazhuang, Hebei; S2_Nanyang, Henan; S3_Shennongjia, Hubei; S4_Bozhou, Anhui; S5_Sanming, Fujian; S6_Zhangshu, Jiangxi; S7_Ganzhou, Jiangxi; S8_Guiyang, Guizhou; S9_Shangluo, Shaanxi; S10_Meizhou, Guangdong, S11_Yulin, Guangxi. Each chromatogram represents the volatile oil profile of a specific batch of *B. pilosa* L. Samples are organized by batch number (S1–S11). The *x*-axis denotes the retention time (minutes), and the *y*-axis represents the detector response (arbitrary units). The peak labeled ‘R’ indicates the retention time of a designated reference control. This figure illustrates the qualitative complexity and potential batch-to-batch variations in the volatile constituents of *B. pilosa* L. oils.

The volatile oil samples extracted from eleven batches of *B. pilosa* collected from different regions were processed and analyzed via GC‒MS. The exported chromatogram and integration data were converted into TXT format and imported into the “Similarity Evaluation System for Traditional Chinese Medicine Fingerprints (2012 Edition)” developed by the National Pharmacopoeia Commission for analysis. Owing to the high peak areas observed in several batches, which resulted in overlapping peaks and difficulties in separation within the software, the signal data, peak heights, and peak areas from all origins were uniformly processed before being imported into the software to generate superimposed chromatograms and a control fingerprint (Figure 1).

Using the chromatogram of the S7 batch of *B. pilosa* volatile oil as a reference standard, a time window of 0.1 min was applied, and the mean method was used for full-spectrum peak matching. After generating the control fingerprint, chromatographic matching was performed to obtain the superimposed chromatogram. The “R” value represents the control fingerprint similarity index, quantifying the degree of similarity between a tested sample and the reference standard. The chromatographic data were standardized on the basis of the stability of the chromatographic peaks and the consistent presence of common peaks in the chromatograms of all 11 batches. In conjunction with the retention time and mass spectrometry data for validation, a total of 43 chromatographic peaks were identified as common peaks (Table 2). The consistency of these 43 common peaks across all samples is the primary basis for the similarity assessment. In the fingerprint comparison (Figure 1), the S7 batch served as the reference standard (R-value = 1.00). While batch S6 exhibited high visual similarity to S7, its calculated R-value was slightly lower due to minor quantitative variations in one or more of these 43 common peaks. Conversely, batch S5, although presenting some noticeable differences in peaks not designated as common, demonstrated a high degree of conformity to S7 with respect to the relative retention times and peak areas of the 43 common peaks, resulting in a similarity index equivalent or comparable to S7. This indicates that the similarity evaluation is driven by the quantitative consistency of the defined common peaks, providing a robust basis for quality control. The establishment of this GC‒MS fingerprint provides a novel approach for controlling the quality of *B. pilosa* medicinal materials.

### 2.3. Heatmap and Cluster Analysis of Volatile Oils from Diverse B. pilosa Origins

**Figure 2:**
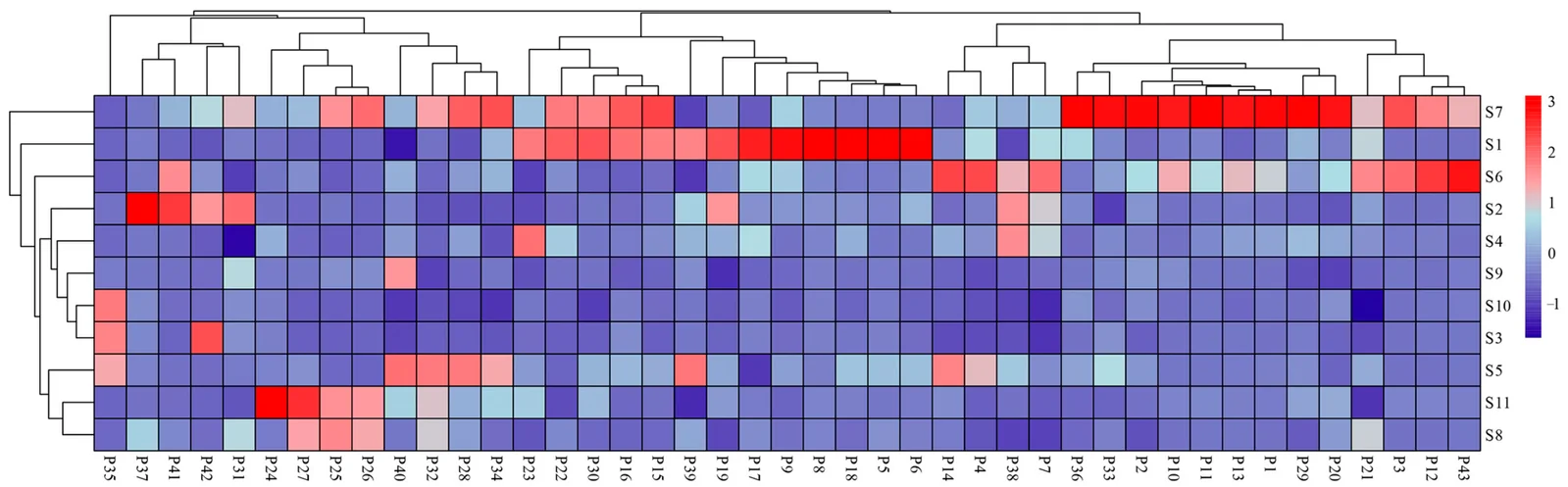
Chemotypic profiling and geographical differentiation of *B. pilosa* L. volatile oils through heatmap and hierarchical cluster analysis. *Note*: This heatmap visually represents the normalized peak intensities of shared volatile compounds detected across 11 distinct batches of *B. pilosa* L. volatile oils. Samples, originating from various Chinese provinces, are hierarchically clustered along the columns based on their compositional similarity. Similarly, volatile compounds (rows) are clustered according to their abundance patterns across the samples. The color intensity reflects the normalized peak abundance of individual compounds, with warmer colors (e.g., red,) indicating higher concentrations and cooler colors (e.g., blue) indicating lower concentrations. This analysis effectively segregates the samples into distinct chemotypes, highlighting significant phytochemical divergence influenced by geographical origin.

GC‒MS fingerprint analysis revealed a high degree of similarity (>0.90) among the volatile oils extracted from eleven batches of *B. pilosa*, indicating substantial consistency in the quality of *B. pilosa* across different geographical origins. On the basis of the fingerprinting analysis conducted within this study, 43 characteristic compounds were identified from the volatile oils of different batches, including P11 (γ-muurolene), P12 (methyl laurate), P17 ((3*Z*)-3-heptadecen-5-yne), P35 (stearic acid), and P37 (stearolic acid). Heatmap and cluster analysis (Figure 2) was performed using the common peak data obtained from the entire profile of these 43 common peaks. Principal Component Analysis (PCA) was also conducted to further visualize sample segregation. The heatmap and cluster analysis revealed that the differential components in *B. pilosa* volatile oils were primarily P11 (γ-muurolene), P12 (methyl laurate), P17 ((Z)-3-heptadecen-5-yne), P35 (stearic acid), and P37 (stearolic acid), indicating their roles in differentiating samples. While the heatmap shows some variation in the P37 component for samples S2 and S8, their grouping in the cluster analysis (Figure 2) signifies a high degree of similarity in their overall volatile oil composition across the 43 common peaks. This overall compositional similarity, rather than homogeneity in any single component, drives their early coalescence in the dendrogram. Cluster analysis grouped the *B. pilosa* samples from different origins into three distinct clusters: (1) S5 (Sanming, Fujian), S3 (Shennongjia, Hubei), and S10 (Meizhou, Guangdong); (2) S1 (Shijiazhuang, Hebei), S9 (Shangluo, Shaanxi), S11 (Yulin, Guangxi), S4 (Haozhou, Anhui), S6 (Zhangshu, Jiangxi), and S7 (Ganzhou, Jiangxi); and (3) S2 (Nanyang, Henan) and S8 (Guiyang, Guizhou). The differential components, particularly P35, played a significant role in separating Group 1 and Group 2. The specific volatile profile of S2 and S8, characterized by unique patterns in components like P37 and others, allowed them to form a distinct third cluster. This overall clustering pattern reflects the influence of geographical origin on the chemical composition of the volatile oils of *B. pilosa*.

### 2.4. Principal Component Analysis of B. pilosa Volatile Oils

**Figure 3:**
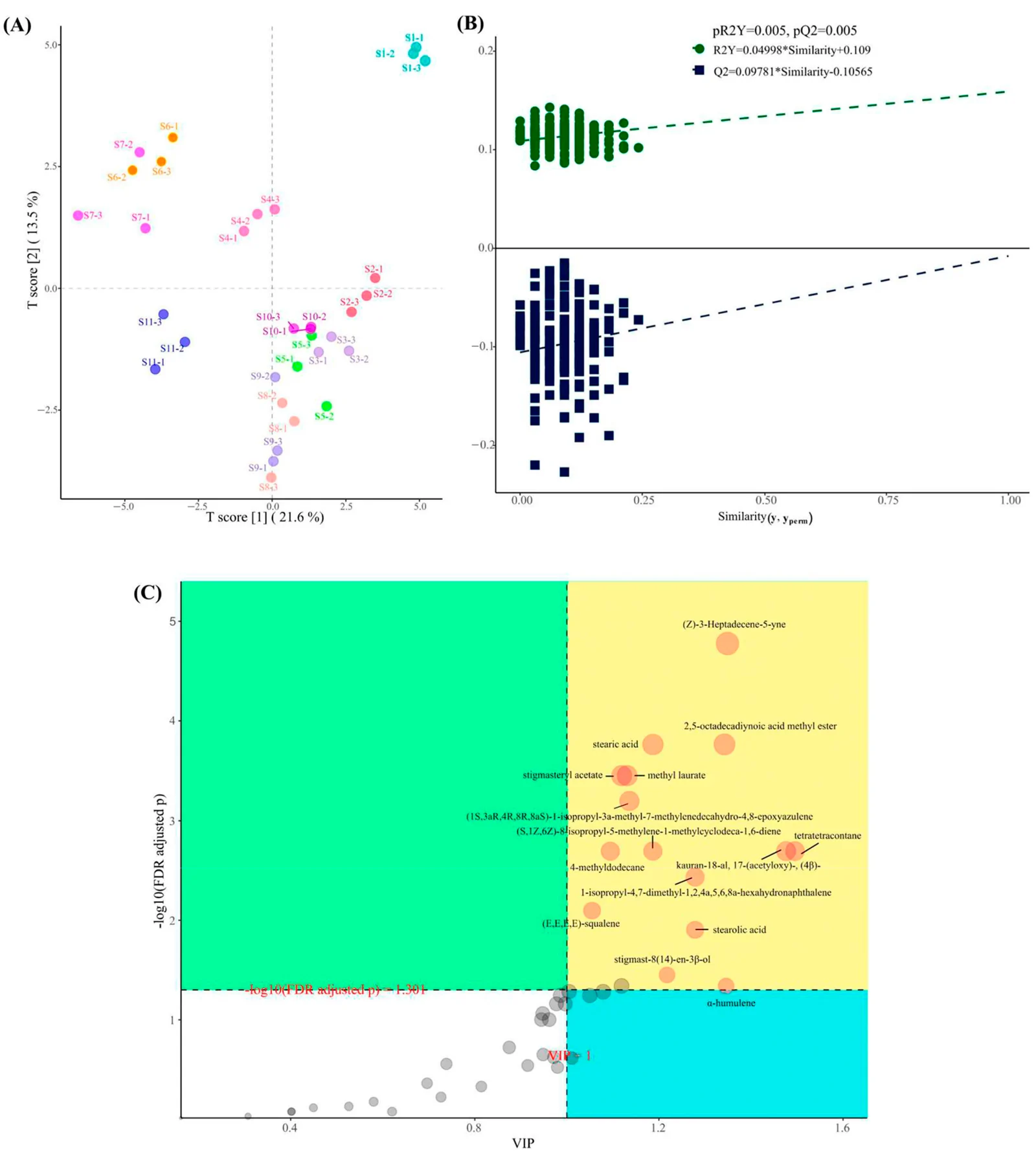
Difference in components and discriminant analysis using partial least squares method in the volatile oil of *B. pilosa* L. (**A**) PLS-DA analysis based on common substances of FDR-corrected features; (**B**) Evaluation of model fitting and prediction ability based on PLS-DA of common substances. (**C**) Variable importance projection (VIP) and significance analysis based on PLS-DA of common substances.

To explore potential patterns and visualize relationships within the volatile oil profiles, Principal Component Analysis (PCA) was performed on the data from 11 batches of *B. pilosa*, using the peak areas of the 43 common peaks as variables. While the analysis identified six principal components with eigenvalues greater than 1, collectively accounting for 95.686% of the variance, it is acknowledged that the statistical robustness of these components may be limited due to the sample size (*n* = 11). This PCA was primarily used for exploratory visualization and to assess the degree to which samples might cluster based on their overall chemical composition. The PCA score plot (Figure 3A) shows that the sample distribution suggests an alignment with the groupings identified in the heatmap and cluster analysis (Figure 2). Samples from Group 1 (S5, S3, S10) appear to aggregate, as do samples from Group 3 (S2, S8), indicating potential distinct chemical profiles. Group 2 (S1, S9, S11, S4, S6, S7) also shows a tendency towards clustering. The detailed breakdown of peak loadings onto individual principal components (Table 3 and Table 4) is provided for transparency but should be interpreted cautiously due to the aforementioned sample size limitations. The calculation of a comprehensive score (F = 0.445 × F1 + 0.222 × F2 + 0.110 × F3 + 0.095 × F4 + 0.049 × F5 + 0.036 × F6) and the subsequent ranking (S1 (0.930) > S5 (0.658) > S8 (0.322) > S2 (0.215) > S9 (−0.030) > S3 (−0.135) > S10 (−0.174) > S4 (−0.318) > S11 (−0.464) > S7 (−0.488) > S6 (−0.517)) is presented as an outcome of this exploratory calculation, rather than a definitive classification (Table 5). Overall, this PCA visualization offers a preliminary perspective on the regional variation in volatile oil composition.

To investigate potential discriminative chemical features among the volatile oil samples from different *B. pilosa* origins, Partial Least Squares Discriminant Analysis (PLS-DA) was employed as an exploratory tool. The analysis utilized the peak areas of the 43 common peaks from the 11 batches. It is crucial to acknowledge the limitations of PLS-DA in this study: with each geographical origin treated as a separate class, there is effectively only one independent sample per class. This scenario is prone to overfitting and pseudoreplication, severely limiting the statistical validity of any definitive class separation. The PLS-DA score plot (Figure 3A) was generated to visualize the sample relationships. In this plot, each data point represents an individual sample batch. While the plot shows some degree of separation among the sample groups, these separations should be interpreted cautiously given the statistical constraints. For instance, the observation that S1 and S11 are positioned in opposite quadrants suggests a pronounced dissimilarity in their volatile oil composition, while the proximity of S6 and S7 indicates a degree of compositional similarity. These observations are noted as preliminary indications of chemical variation. The model fitting and prediction ability evaluation, which yielded pR^2^Y = 0.005 and pQ^2^ = 0.005, are presented but are not considered statistically significant indicators of model effectiveness due to the small sample size per class. The Variable Importance in Projection (VIP) and significance analysis plots (Figure 3C) were used to identify compounds that contributed most to the observed patterns in the PLS-DA model. Compounds such as (Z)-3-heptadecen-5-yne, methyl 2,5-octadecadienoate, stearic acid, methyl laurate, and stearolic acid, identified with VIP scores ≥ 1, are considered putative contributors to the chemical differences observed among the samples. However, due to the aforementioned statistical limitations, these compounds should be regarded as potential discriminators, and their roles require further validation.

### 2.5. Antibacterial Activity of Volatile Oils from Different Geographical Origins Against Pathogenic Bacteria

Under the same concentration conditions, the antibacterial activity of volatile oils from various origins was evaluated via the disc diffusion method against multiple pathogenic bacteria, with ampicillin serving as the positive control. The inhibition zone diameters for the positive control against *E. coli*, *S. aureus*, *P. multocida*, and *S. enterica* were 10.29 ± 0.31 mm, 11.20 ± 0.35 mm, 10.17 ± 0.31 mm, and 12.19 ± 0.44 mm, respectively (Table S4). The antibacterial activity varied significantly across different geographical origins and bacterial strains. For *P. multocida*, samples S6 (12.5 ± 1.46 mm) and S10 (12.93 ± 0.35 mm), originating from Jiangxi Zhangshu and Guangdong Meizhou respectively, displayed the strongest inhibition, exceeding that of several other samples sourced from subtropical regions. While most samples from subtropical humid zones, such as Jiangxi Zhangshu and Guangdong Meizhou, generally showed higher efficacy, the activity of samples like S6 and S10 was particularly prominent amongst this group. Conversely, samples from regions exhibiting weaker activity, such as S7 (6.47 ± 0.17 mm), despite its subtropical origin, and samples from Anhui Bozhou (S4), Henan Nanyang (S2), and Guangxi Yulin (S11) demonstrated relatively lower antibacterial efficacy against *P. multocida*. Notably, sample S2, located in Henan Nanyang (often categorized as a Northern or transitional zone) demonstrated a notable inhibition zone of 6.18 ± 0.08 mm, indicating stronger activity than many southern samples. In contrast to *S. enterica*, samples S10 (10.1 ± 1.49 mm) and S8 (10.17 ± 1.25 mm), originating from Guangdong Meizhou and Guizhou Guiyang, respectively, exhibited the highest inhibition zones. Sample S6 (10.7 ± 0.52 mm) from Jiangxi Zhangshu also showed considerable activity. These southern regions consistently demonstrated larger inhibition zones compared to northern or transitional areas such as Hubei Shennongjia (S3) and Henan Nanyang (S2). For *S. aureus*, samples S6 (11.95 ± 1.23 mm) and S10 (9.38 ± 1.71 mm) from Jiangxi Zhangshu and Guangdong Meizhou again showed the highest activity, reaffirming the superior antibacterial potential from these southern provinces. Regarding *E. coli*, sample S10 (10.42 ± 0.68 mm) from Guangdong Meizhou exhibited the strongest inhibition, followed by S3 (9.88 ± 3.35 mm), S1 (8.62 ± 0.53 mm), and S9 (8.27 ± 0.10 mm). While several of these samples originated from southern China, the varied activity levels underscore the complexity of factors influencing antibacterial efficacy beyond broad geographical classifications. We acknowledge that antibacterial activity was assessed at a single concentration (60 mg/mL) via disk diffusion, which limits quantitative analysis. Specifically, while inhibition zones were observed for all tested samples (excluding the negative control), their sizes varied considerably. Zones with diameters close to the disk size (approximately 6–8 mm) indicate minimal net inhibition beyond the disk’s edge and may reflect limited diffusion or lower antimicrobial potency. Therefore, instead of claiming universal “significant inhibitory effects” for all samples, we focus on identifying those geographical origins and specific samples that demonstrated more pronounced and consistent antibacterial activity across the tested pathogens. The observed variations highlight differences in the composition and efficacy of the volatile oils, with some regions, such as Jiangxi Zhangshu (S6) and Guangdong Meizhou (S10), consistently yielding larger inhibition zones, suggesting a higher concentration or more potent blend of antimicrobial compounds. It is important to note that statistical differences in zone sizes between samples do not always equate to microbiologically relevant or clinically significant activity, especially for smaller zones. Future studies should incorporate MIC and MBC assays to fully characterize antimicrobial potency, establish Q-markers, and determine precise inhibitory/bactericidal concentrations for targeted applications.

**Figure 4:**
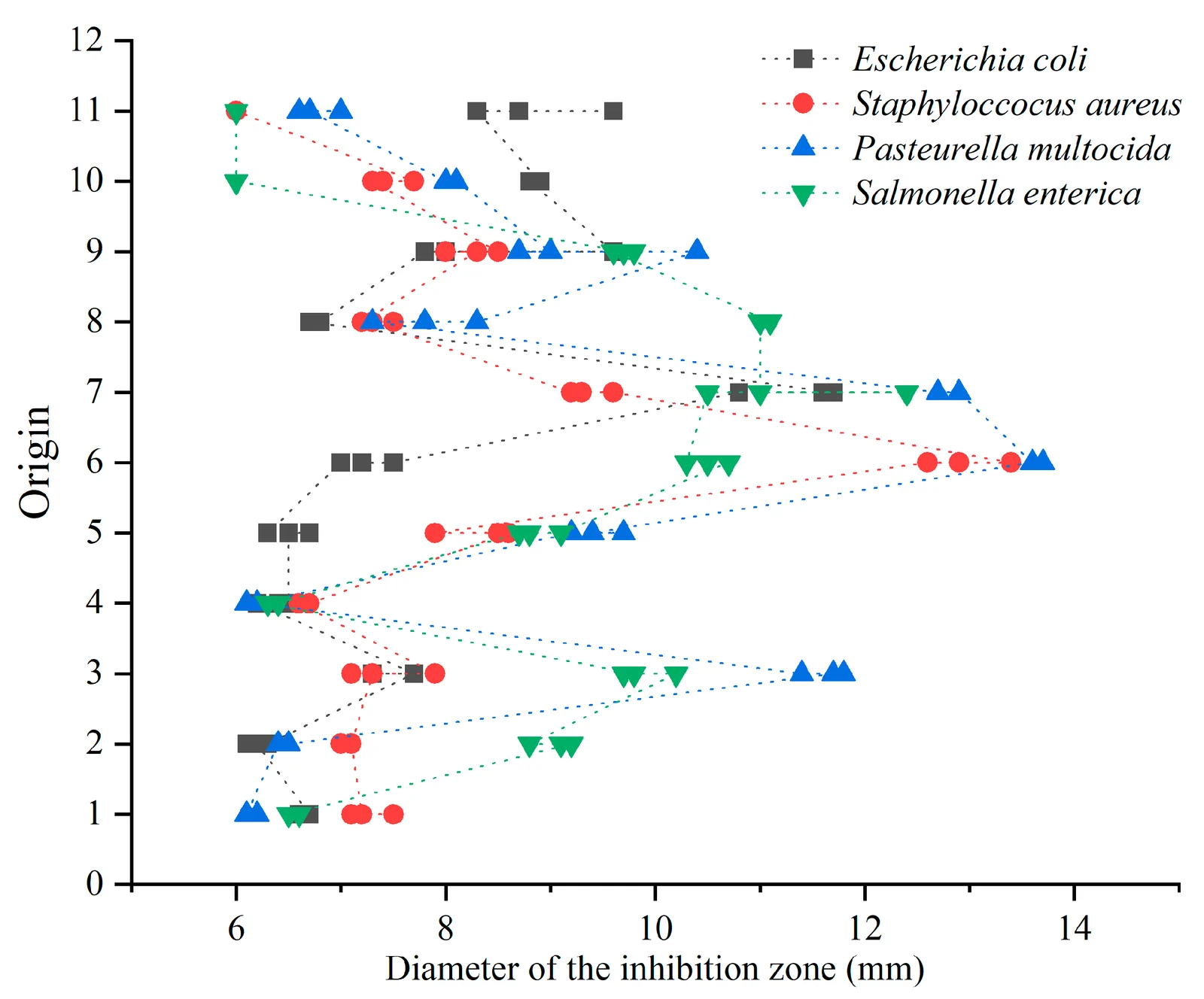
Comparison of the antimicrobial effects of volatile oil from different geographical origins of *B. pilosa* L. against four pathogenic bacteria. *Note*: The ordinal numbers 1 to 11 on the vertical axis represent the following locations, respectively: Guangxi Yulin; Jiangxi Ganzhou; Fujian Sanming; Henan Nanyang; Shaanxi Shangluo; Jiangxi Zhangshu; Guangdong Meizhou; Guizhou Guiyang; Hubei Shennongjia; Hebei Shijiazhuang; Anhui Bozhou.

Overall, the antibacterial effects of the volatile oils from different regions clearly follow a pattern: *S. enterica* > *P. multocida* > *S. aureus* > *E. coli* (Figure 4). This shows that specific locations such as Jiangxi Zhangshu (S6), Guangdong Meizhou (S10), and Guizhou Guiyang (S8) are particularly associated with volatile oils exhibiting significant broad-spectrum antibacterial activity against the tested pathogens. While many southern subtropical regions show a tendency for stronger activity, the presence of actively inhibiting samples like S9 (Shenxi Shangluo) in more northern areas, and the comparatively weaker performance of S7, highlights the influence of specific microclimates, cultivation practices, and genetic variations in the plant, rather than solely relying on generalized geographical zones.

### 2.6. Identification of Candidate Quality Markers for Volatile Oils

All eleven batches of *B. pilosa* volatile oils showed detectable inhibitory effects against *E. coli*, *S. aureus*, *P. multocida*, and *S. enterica*, with notable variations based on geographic origin. The oils derived from Meizhou, Guangdong, exhibited the most potent antibacterial activity against *E. coli* and *P. multocida*, whereas the oils from Jiangxi Zhangshu showed the strongest effects against *S. aureus* and *S. enterica*. To elucidate the chemical basis underlying these antibacterial activities, exploratory gray relational analysis was performed utilizing data from a website (https://www.spsspro.com (accessed on 25 Mar 2025)). The analysis was based exclusively on the inhibition-zone data of the four experimentally tested bacteria, namely *E. coli*, *S. aureus*, *P. multocida*, and *S. enterica*. Specifically, the inhibition-zone diameters of these four bacteria were used as the reference sequences, whereas the peak areas of common components in the fingerprint spectra constituted the comparison sequence. The gray correlation degrees revealed potential associations between chemical constituents and antibacterial activity.

Notably, methyl laurate (P12) exhibited correlation degrees exceeding 0.832 against all four tested bacteria, with particularly high values against *E. coli* (0.976) and *S. aureus* (0.957), indicating a potential key role in antibacterial activity (Table 6). (Z)-3-heptadecen-5-yne (P17) demonstrated correlation degrees above 0.757 across all bacteria, with remarkable associations with *S. aureus* and *P. multocida* (both reaching 0.997), suggesting potential efficacy in combating these strains. Stearic acid (P35) was highly correlated (>0.815), especially with *S. enterica* (0.919), suggesting its importance in inhibiting this pathogen. Similarly, stearolic acid (P37) exhibited correlation degrees exceeding 0.888 for all bacteria, with particularly strong associations with *S. aureus* and *S. enterica*, reaching 0.96. These four compounds were identified through the integration of heatmap visualization, PLS-DA, and GRA. While their strong correlation with antibacterial activity is highlighted, it is crucial to note that GRA, especially with a small dataset (*n* = 11), provides a measure of statistical association and does not establish causation, dose-dependence, or independent biological activity. Therefore, these compounds are proposed as candidate quality markers. They corresponded to common peaks with higher abundances in the heatmap analyses, and PLS-DA identified them as critical contributors to the chemical variability influencing antibacterial activity. By integrating the results from heatmap visualization, PLS-DA, and gray relational analysis, methyl laurate, (Z)-3-heptadecen-5-yne, stearic acid, and stearolic acid are proposed as potential quality markers for *B. pilosa* volatile oils, highlighting their associated antibacterial efficacy. Limitations of this exploratory gray relational analysis include a small dataset (*n* = 11) and the absence of formal statistical validation (confidence intervals, significance tests, robustness/sensitivity analyses, independent validation). While high correlation coefficients suggest statistical association, they do not demonstrate dose dependence, causation, independent activity, or mechanistic relevance. Definitive establishment of these compounds as antimicrobial Q-markers necessitates further investigation. Future studies should individually assess commercial standards via rigorous antibacterial assays (MIC, MBC) and explore synergistic effects to confirm direct contribution, dose dependence, and mechanistic relevance.

## 3. Discussion

This study establishes a preliminary and application-oriented approach for the quality assessment of *B. pilosa* volatile oil, employing SFE-CO_2_ coupled with GC‒MS fingerprinting [19]. Our work addresses a critical gap in the quality evaluation of *Bidens* volatile oil by developing a comparative GC-MS fingerprinting method specifically for this fraction. While prior research has focused on *Bidens* extracts, existing analyses have primarily concentrated on nonvolatile compounds [7]. For instance, a previously established HPLC fingerprint for non-volatiles like rutin and protocatechuic acid revealed substantial inter-source variability, highlighting the inherent challenge in standardizing these components and the consequent neglect of the volatile fraction [20]. Although GC-MS analysis of *Bidens* extracts has been employed in broader pharmacological investigations [21], a dedicated, standardized fingerprint specifically for the quality control of the volatile oil itself has remained elusive. In direct contrast, our study uniquely targets and establishes such a standardized GC-MS fingerprint for the volatile oil fraction of Bidens, offering a useful comparative tool for precise quality assessment. The developed GC‒MS fingerprint successfully identified 43 common peaks across samples from 11 different geographical origins. The dominant chemical classes were esters, fatty acids, and sesquiterpenes. While the majority of samples exhibited remarkable consistency, achieving high similarity indices (0.937–0.995), the sample from Jiangxi Province demonstrated significant deviation, with similarity indices falling below the established threshold [22]. This finding indicates that the Jiangxi sample differed markedly from the majority of the tested samples in its volatile profile; however, the underlying cause could not be determined in the present study because no genetic, agronomic, or environmental data were collected. This observation is consistent with the findings in the results section which indicated notable deviations in the volatile oil content and profile from the Jiangxi Province samples. Nevertheless, acknowledging the limitations discussed in the Data Analysis section, particularly concerning sample size and the potential for pseudoreplication and overfitting in chemometric models, the fingerprint should be interpreted cautiously as a comparative dataset generated under a single analytical framework rather than as a fully validated quality standard. Therefore, while a comparative fingerprint profile was developed for the majority of the *B. pilosa* volatile oil, the Jiangxi sample highlights the need for careful scrutiny of origin-specific variations and potential adulteration or misidentification. While previous GC‒MS analyses of *Bidens* extracts have occurred, they are typically components of broader pharmacological investigations and lack the systematic development of a quality control fingerprint for volatile oils [23]. By establishing a preliminary GC‒MS fingerprint reflecting the characteristic volatile constituents of the examined samples, we provide a concrete analytical basis for batch comparison, authentication of Bidens volatile oil, and the detection of unusual compositional variation. Further methodological validation, including the use of larger sample sets and statistically robust chemometric approaches, will be necessary before this fingerprint can be used as a definitive quality-control standard. This methodology is essential for the reliable utilization of *Bidens*’s abundant resources in TCM and product development, ensuring efficacy and safety on the basis of a defined volatile chemical profile for consistently characterized samples [24]. The current study lays a critical foundation for future pharmacological studies, specifically linking the standardized volatile oil profile of consistent samples to biological activities, while simultaneously highlighting the importance of addressing identified outlier samples to ensure overall quality control.

*E. coli*, *S. aureus*, *P. multocida*, and *S. enterica* are clinically and/or veterinary-important pathogens with distinct ecological niches and disease profiles. *E. coli* and *S. enterica* are major enteric or foodborne pathogens, whereas *S. aureus* is an opportunistic and toxin-producing pathogen, and *P. multocida* is an important animal-associated pathogen with zoonotic relevance. Despite these differences, all four are associated with increasing antimicrobial resistance, underscoring the need for alternative or complementary antimicrobial agents [25]. The increasing antimicrobial resistance reported in these pathogens further underscores the value of investigating plant-derived antimicrobials. In this context, our study evaluated the antibacterial potential of *B. pilosa* volatile oil and examined the variation in activity among samples collected from different geographical origins. Our preliminary assessment via a disc diffusion assay revealed that *B. pilosa* volatile oil possesses significant antibacterial activity against *E. coli*, *S. aureus*, *P. multocida*, and *S. enterica*. This finding aligns with the recognized broad-spectrum antimicrobial properties of many plant-derived volatile oils [26,27], exemplified by studies such as those on *Thymus* spp. oils [28,29]. Crucially, we observed pronounced location-specific variation in the potency of this activity. The volatile oils originating from Guangdong Meizhou, Jiangxi Zhangshu, and Fujian Sanming presented the strongest, broad-spectrum inhibition across the tested strains. In contrast, oils from Guangxi Yuli, Henan Nanyang, Jiangxi Ganzhou, and Anhui Bozhou showed markedly weaker activity. For instance, while oils from Guangdong Meizhou inhibited all tested bacteria, the oil from Henan Nanyang displayed limited inhibition solely against *P. multocida*. This pattern indicates that the geographical origin of the sampled materials was associated with differences in the observed antibacterial activity of the volatile oil [30]. However, because the present study did not measure climate variables, collect soil samples, determine selenium concentrations or other soil nutrient parameters, record agronomic information, perform genetic identification, or conduct controlled cultivation experiments, the specific causes underlying these origin-dependent differences cannot be determined from the current dataset. Accordingly, the present results support only the conclusion that antibacterial activity differed among samples from different geographical origins, whereas the mechanistic basis of this variation remains unresolved. Previous studies have shown that environmental and edaphic factors can influence plant secondary metabolism and thereby alter the accumulation of bioactive constituents [31,32,33,34,35]. In this context, such research provides a possible interpretive framework for understanding origin-related chemical and biological variation in medicinal plants. However, in the absence of direct measurements, these factors should be regarded only as plausible hypotheses rather than demonstrated explanations for the present findings. Likewise, although differences were observed among samples collected within Jiangxi Province, these patterns should not be interpreted as direct evidence for the effects of selenium-rich acidic soils, heavy clay, low organic matter, or genetic/varietal divergence, because none of these variables was directly examined in this study. Instead, the intra-provincial variation observed here indicates that samples from different localities may differ substantially in volatile-oil composition and antibacterial performance, but the relative contributions of environmental conditions, soil properties, cultivation practices, harvest stage, post-harvest handling, and genetic background remain to be clarified. Furthermore, our earlier findings regarding varietal confusion and inconsistent quality observed in some samples from Jiangxi Province (specifically samples 6 and S7) warrant careful consideration. While soil properties may play a role in the difference between Zhangshu and Ganzhou, the distinct variability observed in other Jiangxi samples suggests that genetic or varietal differences at the species or subspecies level could also be a significant driver of bioactivity divergence in certain locations.

Currently, spectrum–effect correlation is an important method for screening quality markers of traditional Chinese medicine (TCM) [36]. A commonly used analytical method is gray relational analysis (GRA). GRA is a multifactor statistical analysis method that can explore the complex correlations between various variables and factors during the development of a system [37]. This study screened candidate antimicrobial quality markers (Q-markers) for *B. pilosa* volatile oil through multidimensional spectrum‒effect relationship analysis, linking chemical composition to antibacterial efficacy. The selection of spectrum‒effect relationship analysis aligns with the established paradigm for identifying Q-markers in TCM, which necessitates the correlation of specific chemical constituents with pharmacological activity [38]. Our approach aligns with established methodologies used for other medicinal plant volatile oils, such as those employed for identifying antimicrobial Q-markers in *Zanthoxylum armatum* DC. [39]. This methodological consistency underscores the validity and increasing adoption of spectrum‒effect analysis for standardizing bioactive volatile oils. Our findings, which demonstrated significant geographical variation in both chemical profiles and antibacterial potency, provided the essential foundation for this correlative analysis. Gray relational analysis revealed that four constituents showed a strong association with the observed broad-spectrum antibacterial activity against *E. coli*, *S. aureus*, *P. multocida*, and *S. enterica*. However, it should be emphasized that GRA-based spectrum‒effect correlation only reflects statistical association between chromatographic peaks and antibacterial activity, and does not by itself establish direct biological causation. Therefore, these compounds should be interpreted as potential or candidate antimicrobial Q-markers rather than definitively validated Q-markers. For instance, methyl laurate and stearic acid were identified as compounds strongly associated with oil bioactivity. Methyl laurate has been identified as a key component in *Lonicera japonica* essential oil due to its antibacterial properties [40]. Additionally, studies have demonstrated that lauric acid and its derivatives exhibit potent antifungal activity, primarily by disrupting plasma membrane integrity, which results in leakage of the cellular contents and increased membrane conductivity. The lipophilic nature of methyl laurate likely facilitates its enhanced penetration into bacterial membranes, thereby amplifying its disruptive effects [41]. While direct mechanistic studies on the intrinsic antibacterial activity of stearic acid in volatile oils are relatively limited, relevant indirect evidence has been presented. Research has demonstrated that modification with stearic acid can significantly enhance antibacterial efficacy by achieving up to 99% inhibition against *E. coli* and *S. aureus*, without compromising other properties of functional bacterial cellulose films [42,43]. This finding indicates a potential synergistic or intrinsic antimicrobial role for stearic acid within such systems. Similarly, (Z)-3-heptadecen-5-yne and stearolic acid were found to be associated with antimicrobial effects, potentially through disruption of microbial membrane integrity and inhibition of metabolic enzyme activity [26,44]. From a structure–activity relationship perspective, the antibacterial activity of polyacetylenes may be related to their conjugated triple-bond systems, which can enhance lipophilicity and reactivity, disrupt bacterial membrane integrity, and increase permeability [45]. Thus, compounds such as (Z)-3-heptadecen-5-yne and stearolic acid may contribute to the antibacterial activity of *B. pilosa* volatile oil through membrane perturbation, although this remains to be confirmed experimentally. Importantly the antibacterial activity of volatile oils is often governed by the overall chemical matrix rather than by a single dominant compound. Major polyacetylenes and minor sesquiterpenes may act independently, additively, or synergistically, making the contribution of each constituent difficult to interpret in isolation. Therefore, the candidate Q-markers identified in this study may represent either directly active compounds or components involved in cooperative interactions within the volatile matrix. GRA could not distinguish whether methyl laurate, stearic acid, (Z)-3-heptadecen-5-yne, and stearolic acid act independently or through additive or synergistic effects with other constituents. Accordingly, these compounds should be considered candidate markers of the active volatile matrix rather than definitive evidence of single-compound efficacy.

Importantly, the current study did not include antibacterial testing of isolated commercial standards of these key compounds individually. Therefore, their independent contributions to the observed antibacterial activity cannot yet be conclusively confirmed. To firmly establish these compounds as antimicrobial Q-markers, further studies should evaluate commercially available standards of the major polyacetylenes and terpenes using individual antibacterial assays, such as inhibition zone, MIC, and/or MBC determinations, and compare their activities with those of the whole volatile oil. Such experiments would help distinguish whether the identified compounds are truly active principles, merely correlated constituents, or contributors within a multi-component interaction network. combination assays using defined mixtures of major polyacetylenes and representative minor sesquiterpenes should be performed to determine whether their interactions are independent, additive, or synergistic. Approaches such as checkerboard microdilution, fractional inhibitory concentration index (FICI) analysis, or response-surface modeling would be particularly useful for this purpose. In addition, because volatile oils often exert their biological effects through additive or synergistic interactions among major and minor constituents, future studies should also assess potential synergistic effects among the identified candidate compounds. Accordingly, the compounds identified in this study are more appropriately described as candidate antimicrobial quality markers supported by spectrum‒effect correlation analysis. The associations identified in this study provide a targeted basis for the standardization and sustainable development of *B. pilosa* resources, but they should not be interpreted as definitive evidence of individual compound-level causation without further experimental validation using isolated standards.

## 4. Materials and Methods

### 4.1. Preparation of B. pilosa Volatile Oil

Eleven batches of *B. pilosa* samples were collected from key traditional Chinese medicine-producing areas across China (Figure S1): S1_Hebei Shijiazhuang, S2_Henan Nanyang, S3_Hubei Shennongjia, S4_Anhui Bozhou, S5_Fujian Sanming, S6_Jiangxi Zhangshu, S7_Jiangxi Ganzhou, S8_Guizhou Guiyang, S9_Shaanxi Shangluo, S10_Guangdong Meizhou, and S11_Guangxi Yulin. Detailed geographic and environmental information for all sampling sites, including location, climate type, annual rainfall, and GPS coordinates, is provided in Table S1. For all collections, the aerial parts (consisting of leaves, stems, and inflorescences with flowers and developing seeds) of *B. pilosa* were harvested at the full flowering stage, characterized by the presence of mature flowers and seeds but before significant senescence, and all samples were collected during the same period from August to October. To minimize variation associated with plant developmental stage and harvest season, all samples were collected at the same phenological stage and within a comparable collection window. The collected samples were immediately transported to the laboratory. All collected materials were identified as *B. pilosa* L. (Asteraceae) based on the macroscopic morphological characteristics of the aerial parts, inflorescences, and seeds, with reference to the Flora of China and taxonomic identification keys for the genus Bidens by Professor Xianghai Kong, Longyan University (Table S2).

Extraction of volatile extracts (VOs) [46]: Fresh aerial parts (leaves, stems, and inflorescences) of *Bidens pilosa* L. were washed, air-dried under controlled conditions (25 °C, 40–55% RH) for 7–10 days to achieve constant weight, and then ground into fine powder (<150 mesh) with intermittent cooling to prevent thermal degradation. VOs were extracted using a supercritical CO_2_ extraction system (HA121-50-01, Jiangsu Nantong Hu’an Supercritical Extraction Co., Ltd., Nantong, China). A total of 150 g of powder was loaded into the extraction vessel with 12 g of fresh 95% ethanol aqueous solution (*v*:*v*), degreased cotton (4.22 g), and filter paper. The system was operated at an extraction temperature of 45 °C, Separation I at 40 °C, and Separation II at 30 °C. CO_2_ was introduced gradually to equalize pressure between the storage tank and extraction chamber. The pump frequency was set to 15 Hz, and the extraction pressure was raised to 33.50 MPa. A carefully controlled pressure gradient was maintained: the pressure in the extraction vessel was 33.50 MPa, and the pressures in Separator I and Separator II were set to 8.60 MPa and <6.0 MPa, respectively, to facilitate efficient compound separation. The collection of the volatile oil extract occurred at 9 MPa, representing the pressure required for liquid collection at the given operating temperatures. The extraction was performed for 2.5 h. Upon completion, the system was depressurized sequentially: the CO_2_ inlet valve, pump, and cylinder valve were closed; and heating was deactivated after the pressure in Separator I dropped to 3.0 MPa. VOs from both separators were collected at 9 MPa.

Lyophilization of volatile extract: The obtained *B. pilosa* volatile oil extract (Figure S2) was weighed, and its volume was measured. The samples were sealed and stored at −80 °C for preservation. Each sample from different regions was extracted in triplicate, and the extraction yield of the volatile extract (%) was calculated by taking the average value (Table 1). The volatile extract was stored at −80 °C for at least 24 h to ensure complete solidification. The lyophilizer and vacuum pump (ALPH R 2-4LD PLUS, Beijing Bolixing Instruments Co., Ltd., Beijing, China) were then activated. The system was preheated for 20 min in warm-up mode to prevent the solvent vapor from damaging the oil. The frozen samples were covered with perforated sealing films and placed in a crucible equipped with a sample rack, which was then covered with a transparent acrylic lid. The vacuum pump was started, and the drying process was conducted in main drying mode for 12 h. During the main drying phase, the shelf temperature was maintained at −20 °C, and the vacuum was applied until the system reached the set temperature and achieved the desired level of dryness. After drying, the vacuum pump was gradually shut off, and the samples were slowly depressurized and removed. The obtained *B. pilosa* volatile oil extract, which appeared as a paste-like residue after lyophilization, was carefully weighed and transferred to a moisture-proof, sealed bag. The samples were labeled accordingly and stored at −80 °C to preserve bioactivity for subsequent analyses. The presence of some non-volatile compounds, such as fatty acids, in the analyzed volatile oil fraction is acknowledged and is a common observation in essential oil analysis, potentially arising from co-extraction of minor non-volatile components. The primary purpose of lyophilization in this context was to remove residual moisture and water from the CO_2_ extraction process, allowing for accurate weighing of the volatile oil, rather than complete desiccation of all volatile components.

### 4.2. Analysis of Volatile Oil Components via Gas Chromatography‒Mass Spectrometry (GC‒MS)

A 0.05 g sample was accurately weighed into a 2 mL Eppendorf tube. Then, 1mL of prechilled n-hexane extraction solvent was added, and the mixture was vortexed for 30 s. The steel beads were added and homogenized via a grinder at 40 Hz for 4 min, followed by sonication in an ice–water bath for 5 min (repeated 3 times). The sample was centrifuged at 12,000 rpm (13,800× *g*, radius 8.6 cm) at 4 °C for 15 min. A total of 150 μL of the supernatant was carefully transferred into a vial. Eighty microliter aliquots from each sample were mixed to create a quality control (QC) sample, which was analyzed on the instrument sequentially.

GC‒MS analysis was conducted via a SHIMADZU GC‒2020 (Shimadzu Corporation, Kyoto, Japan) gas chromatograph coupled with a mass spectrometer [46]. The analysis employed a DB-5MS capillary column with dimensions of 30 m in length, 0.25 mm inner diameter, and a 0.25 μm film thickness. An aliquot of 1 μL of the sample was injected in splitless mode. Helium served as the carrier gas, with a front inlet purge flow rate of 3 mL/min, and the column flow rate was maintained at 1 mL/min. The initial oven temperature was set to 50 °C, held for 1 min, increased to 310 °C at a rate of 8 °C/min, and maintained at 310 °C for an additional 11.5 min. The injection port, transfer line, and ion source were maintained at 280 °C, 280 °C, and 200 °C, respectively. Electron ionization was performed at an energy of −70 eV. Mass spectral data were acquired in full-scan mode over a *m*/*z* range of 50–500, with a data collection rate of 12.5 spectra per second, starting after a solvent delay of 7.2 min. Compound identification was achieved through a dual approach of retention index (RI) determination and mass spectral matching. Prior to sample analysis, a homologous series of n-alkanes (C9–C25) were injected under identical GC-MS conditions. Their retention times were used to calculate Kovats retention indices. Subsequently, retention indices for all detected peaks in the *B. pilosa* volatile extract samples were computed using the established equation for linear programmed temperature vaporization gas chromatography. Compounds were tentatively identified by comparison of mass spectra with the NIST 2014 library and by retention index matching. A compound was assigned when the spectral similarity was ≥90% and the RI deviation was within ±10 units of the reference value. For structurally similar isomers or ambiguous spectral matches, confirmation involved direct comparison with reported literature data.

### 4.3. Determination of the Antibacterial Activity of the Volatile Oil [47]

Thirty-six grams of LB agar powder was accurately weighed and dissolved in 1 L of distilled water. The mixture was stirred and heated to boiling until completely dissolved, dispensed into Erlenmeyer flasks, sealed, and sterilized in an autoclave at 121 °C for 20 min. After cooling, the sterilized agar was poured into sterile Petri dishes (90 mm diameter) and allowed to solidify. Separately, 21.00 g of LB broth powder was dissolved in 1 L of distilled water, stirred, and boiled until fully dissolved. The broth solution was distributed into Erlenmeyer flasks, sealed, and sterilized at 121 °C for 20 min in an autoclave.

Bacterial cultures—*Escherichia coli* (CMCC 44103), *Staphylococcus aureus* (CMCC 26003), *Pasteurella multocida* (CVCC 1685), and *Salmonella enterica* (CICC 21513)—were individually inoculated into LB broth for liquid culture. The cultures were incubated with shaking at 37 °C for 6 h. The bacterial suspensions were then diluted with sterile LB broth to achieve a final concentration of 1 × 10^5^ CFU/mL. This concentration was carefully adjusted to ensure consistent and vigorous growth of confluent bacterial lawns on the LB agar plates, which is essential for reliable comparative screening of the volatile extracts’ antibacterial activity.

A total of 0.06 g of *B. pilosa* volatile extract was accurately weighed and dissolved in 1% Tween-80 solution to prepare a stock solution of 60 mg/mL. Specifically, the volatile extract was precisely measured and mixed with Tween-80 to ensure complete solubilization. Additionally, 10 mg of sodium ampicillin was accurately weighed and dissolved in a 1% Tween-80 solution to produce a positive control solution with a concentration of 10 mg/mL. All the solutions were prepared under aseptic conditions and stored at appropriate temperatures until use. For the purpose of this study, focusing on the geographical variation in antibacterial activity, the antibacterial activity of the volatile extract was evaluated via the disk diffusion method at a single concentration (60 mg/mL) to facilitate a comparative screening of multiple samples. While the disk diffusion assay provides an indication of the presence and relative antibacterial potency, Minimum Inhibitory Concentration (MIC) and Minimum Bactericidal Concentration (MBC) values were not determined in this study, as the primary focus was on the comparative screening of volatile extracts across different geographical origins. The antibacterial activity of the volatile extract was evaluated via the disk diffusion method by measuring the diameter of the inhibition zones. The antibacterial activity of the volatile extract was evaluated via the disk diffusion method (Figure S3). For each bacterial species (*E. coli*, *S. aureus*, *P. multocida*, and *S. enterica*), three independent biological replicates were performed. In each replicate, 100 μL of the adjusted bacterial suspension (approximately 1 × 10^5^ CFU/mL) was uniformly spread onto separate LB agar plates (90 mm diameter) in a biosafety cabinet to produce confluent bacterial lawns. Subsequently, in each replicate, a sterile filter paper disc (6 mm diameter) was aseptically placed onto the surface of one plate designated for the *B. pilosa* volatile extract, another plate for the positive control (10 mg/mL ampicillin sodium), and a third plate for the negative control (1% Tween-80). This experimental setup ensured that each plate within a replicate tested only a single treatment (i.e., one volatile oil sample, the positive control, or the negative control), with the biological replicates serving to account for plate-to-plate variability and ensure the robustness of the comparative analysis. A volume of 20 μL of the respective test solution was applied to each disc. Consequently, the loading amounts per disk were approximately 1.2 mg for the volatile oil extract and 200 µg for ampicillin. While these distinct loading amounts necessitate caution when directly comparing the magnitude of inhibition zones between the extract and ampicillin, the assay remains effective for evaluating the presence and relative antibacterial activity of the volatile extract and for comparative screening across different samples. It is acknowledged that the physical diffusion of highly lipophilic extracts and long-chain fatty compounds through agar can be variable and compound-dependent, potentially influencing the observed inhibition zone sizes. The results presented thus reflect the antibacterial activity of the diffusible components under the specified assay conditions. For the negative control (1% Tween-80), no inhibition zones were observed around the discs, confirming the absence of any solvent-induced antibacterial effect. Following disc placement, all plates were inverted and incubated at 37 °C for 18–24 h. The diameters of the inhibition zones were measured via a digital caliper. The mean zone diameter and standard deviation were calculated from the results of the three independent replicates.

### 4.4. Establishment of a GC‒MS Fingerprint Profile for Volatile Extracts

Gas chromatography‒mass spectrometry (GC‒MS) analysis was performed on eleven batches of volatile extract l samples extracted from *B. pilosa*. The GC‒MS data were processed via a postrun analysis workstation, where peak areas were integrated for qualitative analysis. All component mass spectra were subsequently identified through computer-assisted library searches against the NIST14 and MASS14s standard spectral libraries. The preliminary identification of volatile extract constituents was based on spectral matching. Relative quantification of individual components was performed using the peak area normalization method. This approach provides an estimation of the relative abundance of compounds, enabling the comparative analysis of chemical profiles and the construction of a comparative GC-MS fingerprint. Following data integration, the chromatographic and integrated data were organized and exported to a text (*.txt) file. During this process, the “Encoding” option was set to “ANSI,” and all spaces were removed to ensure data consistency. The complete txt file was then imported into the “Chromatographic Fingerprint Similarity Evaluation System” developed by the Chinese Pharmacopoeia Commission (version 2012.130723). The software was used to analyze the GC‒MS data, establish the fingerprint profile of *B. pilosa* volatile extract, and calculate the similarity coefficients among samples. For fingerprint construction, peaks were considered as candidate common peaks only when they were consistently detected in all 11 batches, showed relatively stable retention times, and could be supported by mass spectral identification. Chromatographic alignment was performed in the Similarity Evaluation System by using batch S7 as the reference chromatogram with a retention-time matching window of 0.1 min, followed by full-spectrum peak matching using the mean method. To improve data reliability, the automatically integrated peaks were manually inspected, and peak boundaries were corrected when necessary in cases of peak overlap, baseline drift, or obvious integration error. It should be noted that this study focused on comparative fingerprint establishment under the same analytical conditions; formal analytical validation, including repeatability, inter-day precision, and stability evaluation, was not included in the present work.

### 4.5. Data Analysis

The experimental data were analyzed via SPSS version 18.0 (SPSS Inc., Chicago, IL, USA). Antibacterial activity (inhibition zone diameters) was analyzed per bacterial strain using one-way ANOVA (*p* < 0.05). Data normality (Shapiro–Wilk) and variance homogeneity (Levene’s) were confirmed, followed by Tukey’s HSD post hoc test for significant differences. Means of three technical replicates per oil-strain combination were used. This strain-specific, origin-based comparison, while limited by technical replicates, directly assessed inhibitory effects. Complex interactions could be explored with two-factor ANOVA or multiple testing correction, but our focus remained on these direct comparisons. The 43 common peaks obtained from the fingerprint chromatograms were used for chemometric analyses. Heatmap visualization and hierarchical clustering analysis were performed using a data analysis platform (https://hiplot.com.cn/home/index.en.html (accessed on 15 April 2025)) to explore compositional relationships and potential groupings based on the relative abundance of these common peaks. Principal component analysis (PCA) was conducted with SPSS 18.0 as an exploratory tool to visualize sample relationships. Components with eigenvalues greater than 1 were retained for interpretation. Given the limited number of independent samples (*n* = 11, with each geographical origin treated as a single observation unit for these analyses) and the higher number of variables (43), this approach is primarily for data dimensionality reduction and visualization of trends, rather than definitive statistical classification. Partial least squares discriminant analysis (PLS-DA) was performed via an online platform (https://www.bioincloud.tech/ (accessed on 19 May 2025)). This analysis was conducted to investigate potential differences in chemical profiles among the geographical origins. As noted, treating each geographical origin as a separate class with only one sample per class inherently limits the statistical power and generalizability of the findings. Therefore, the PLS-DA results, particularly regarding classification of separate origins, should be interpreted as suggestive of compositional variations rather than definitive class separations. Significant differentially abundant metabolites were identified with variable importance in projection (VIP) scores ≥ 1 and a significance threshold −log_10_ (*p* value) > 1.301 (corresponding to *p* < 0.05). These indicators are derived from a model with inherent statistical limitations, and the PLS-DA results, particularly the classification of separate origins, should be considered as exploratory due to the limited number of samples per class. To explore the relationship between common peaks and antibacterial activity, Pearson correlation analysis was performed on the basis of the spectral peak areas and inhibition zone diameters against *E. coli*, *S. aureus*, *S. enterica*, and *P. multocida*. The common peaks, labeled P1–P43, served as the comparison variables, whereas the inhibition zone diameters served as the reference sequence. Furthermore, gray relational analysis (GRA) was applied to evaluate the degree of association between each common peak and antibacterial activity. The gray relational grades were calculated, ranked in decreasing order, and used to construct an ordered relational sequence. This sequence provides an intuitive reflection of the influence of each fingerprint component on antibacterial activity, with higher relational grades indicating a stronger contribution of the corresponding peaks to the observed antibacterial effects. GRA was chosen as it provides a measure of correlation strength, which is less sensitive to sample size limitations compared to classification-based models.

## 5. Conclusions

This study presents an integrated approach for assessing the quality and evaluating the bioactivity of *B. pilosa* L. volatile oil, which is characterized by three key innovations. A preliminary GC‒MS fingerprinting protocol was developed to identify 43 characteristic peaks, mainly esters, fatty acids, and sesquiterpenes, with high inter-batch similarity, thereby providing a comparative analytical basis for the quality evaluation of *B. pilosa*. Bioactivity analysis revealed significant antibacterial activity against pathogens such as *E. coli*, *S. aureus*, *P. multocida*, and *S. enterica*, with potency substantially influenced by geographical origin; volatile oils from subtropical monsoon regions (Meizhou, Zhangshu, Sanming) exhibited superior efficacy. Spectrum–effect relationship analysis identified four candidate quality markers: methyl laurate and stearic acid, (3*Z*)-3-heptadecen-5-yne and stearolic acid. These findings provide a scientific foundation for standardized production of *B. pilosa* volatile oil on the basis of verifiable candidate chemical markers. Mechanistic studies focusing on the unique antimicrobial actions of alkynic compounds, coupled with isolation and MIC determination of the candidate Q-markers, are necessary to validate their individual and synergistic roles, ultimately facilitating the clinical translation of geographically optimized *B. pilosa* extracts. This research bridges the gap between traditional use and evidence-based application, offering a model for the standardized quality control of plant-derived antimicrobials.

## Figures and Tables

**Table 1:** Oil yield of *B. pilosa* L. essential oil prepared from 11 different regions in China.

Origin	Essential Oil Yield (%)
S1	1.13 ± 0.0020 ^e^
S2	0.95 ± 0.0027 ^f^
S3	1.30 ± 0.0024 ^c^
S4	1.32 ± 0.0028 ^c^
S5	1.24 ± 0.0037 ^d^
S6	1.20 ± 0.0090 ^d^
S7	2.37 ± 0.0260 ^b^
S8	0.83 ± 0.0016 ^g^
S9	2.94 ± 0.0164 ^a^
S10	1.35 ± 0.0048 ^c^
S11	2.99 ± 0.0049 ^a^

*Note:* S1_Shijiazhuang, Hebei; S2_Nanyang, Henan; S3_Shennongjia, Hubei; S4_Bozhou, Anhui; S5_Sanming, Fujian; S6_Zhangshu, Jiangxi; S7_Ganzhou, Jiangxi; S8_Guiyang, Guizhou; S9_Shangluo, Shaanxi; S10_Meizhou, Guangdong, S11_Yulin, Guangxi. Data are expressed as mean ± standard deviation (*n* = 3). Different superscript letters indicate statistically significant differences (*p* < 0.05) among the means of different regions according to Tukey’s HSD test.

**Table 2:** Identification of common volatile oil components from eleven batches of *B. pilosa* by GC-MS analysis.

ID	Retention Time	KI (Calculated Kovats)	RI #(HP-5MS)	Component *	Molecular Formula	CAS	Confidence Level
P1	8.667	1122	-	cis-cyclohexane-1,4-dimethanol diacetate	C_12_H_20_O_4_	6308-18-5	Level 3
P2	9.063	1126	1133	cis-verbenol	C_10_H_16_O	473-67-6	Level 2
P3	12.114	1259	1260	4-methyldodecane	C_13_H_28_	6117-97-1	Level 2
P4	13.275	1384	1386	β-bourbonene	C_15_H_24_	5208-59-3	Level 2
P5	13.875	1413	1419	caryophyllene	C_15_H_24_	87-44-5	Level 2
P6	14.458	1448	1453	α-humulene	C_15_H_24_	6753-98-6	Level 2
P7	14.802	1457	1469	β-ionone	C_13_H_20_O	79-77-6	Level 2
P8	14.867	1460	-	(S,1Z,6Z)-8-isopropyl-5-methylene-1-methylcyclodeca-1,6-diene	C_15_H_24_	23986-74-5	Level 2
P9	15.105	1467	1452	1-isopropyl-4,7-dimethyl-1,2,4a,5,6,8a-hexahydronaphthalene	C_15_H_24_	483-75-0	Level 2
P10	15.233	1474	1481	β-bisabolene	C_15_H_24_	495-61-4	Level 2
P11	15.363	1480	1489	γ-muurolene	C_15_H_24_	30021-74-0	Level 2
P12	15.438	1498	1488	methyl laurate	C_13_H_26_O_2_	111-82-0	Level 2
P13	15.692	1531	1525	dihydroactinidiolide	C_11_H_16_O_2_	17092-92-1	Level 2
P14	15.969	1551	1560	(±)-trans-nerolidol	C_15_H_26_O	40716-66-3	Level 2
P15	16.05	1589	1561	(-)-caryophyllene oxide	C_15_H_24_O	1139-30-6	Level 2
P16	16.233	1591	1573	mintoxide	C_15_H_24_O	88395-47-5	Level 3
P17	16.467	1595	-	(*Z*)-3-heptadecene-5-yne	C_17_H_3_O	74744-55-1	Level 3
P18	16.613	1605	1599	salvial-4(14)-en-1-one	C_15_H_24_O	73809-82-2	Level 2
P19	16.872	1614	1606	humulene epoxide II	C_15_H_24_O	19888-34-7	Level 2
P20	16.958	1622	-	2,5-octadecadiynoic acid methyl ester	C_19_H_30_O_2_	57156-91-9	Level 3
P21	17.134	1625	1631	isospathulenol	C_15_H_24_O	88395-46-4	Level 4
P22	17.221	1632	-	(+)-8(15)-cedren-9-ol	C_15_H_24_	41432-70-6	Level 4
P23	17.324	1638	-	9h-fluorene	C_13_H_10_	86-73-7	Level 4
P24	17.404	1658	-	aromadendrene oxide II	C_15_H_24_O	85710-39-0	Level 4
P25	17.513	1667	-	1-(2,4-dimethylphenyl)-2-methylpropan-1-one	C_12_H_16_O	23351-72-6	Level 4
P26	17.62	1730	-	octadec-9-yne	C_18_H_34_	35365-59-4	Level 4
P27	18.062	1735	-	1,4-cycloheptadiene,6-butyl-	C_11_H_18_	22735-58-6	Level 4
P28	19.425	1758	-	hexadecane	C_16_H_34_	544-76-3	Level 4
P29	19.811	1825	-	(*Z*)-14-methylhexadec-8-enal	C_17_H_32_O	60609-53-2	Level 4
P30	19.901	1844	1840	neophytadiene	C_20_H_38_	504-96-1	Level 3
P31	19.983	1846	1838	phytone	C_18_H_36_O	502-69-2	Level 2
P32	20.226	1889	1967	benzyl benzoate	C_14_H_22_O_4_	120-51-4	Level 2
P33	20.691	1908	1897	ethyl pentadecanoate	C_17_H_34_O_2_	41114-00-5	Level 3
P34	20.988	1955	-	kauran-18-al, 17-(acetyloxy)-, (4β)-	C_22_H_34_O_3_	41756-46-1	Level 3
P35	21.94	2102	2188	stearic acid	C_18_H_36_O_2_	57-11-4	Level 3
P36	23.186	2120	2116	methyl α-linolenate	C_19_H_32_O_2_	301-00-8	Level 2
P37	24.311	2162	2145	stearolic acid	C_18_H_32_O_2_	506-24-1	Level 3
P38	26.949	2170	-	octadecanaldehyde	C_18_H_36_O	638-66-4	Level 4
P39	27.527	2320	-	nonadecane	C_19_H_40_	629-92-5	Level 4
P40	28.5	2482	-	behenic acid ethyl ester	C_24_H_48_O_2_	5908-87-2	Level 4
P41	32.362	2552	-	stigmasteryl acetate	C_31_H_50_O_2_	4651-48-3	Level 4
P42	35.555	2687	-	stigmast-8(14)-en-3β-ol	C_29_H_50_O	18069-99-3	Level 4
P43	41.646	2784	-	(*E*,*E*,*E*,*E*)-squalene	C_30_H_50_	111-02-4	Level 3

Note: * indicated that compounds were considered positively identified when the mass-spectral similarity exceeded 0.95 and the calculated RI was within ±10 units of the literature value; # indicated that derived from Data from NIST Standard Reference Database 69: NIST Chemistry Web-Book. Confidence levels (MSI Levels) were assigned as follows: Level 1_Identified by co-injection with authentic standard (not applicable in this study). Level 2_Tentatively identified by matching mass spectrum (similarity ≥ 90%) and RI value (ΔRI ≤ ±10). Level 3_Tentatively identified by mass spectrum similarity (≥90%) but without RI validation or ΔRI > ±10. Level 4_Unknown compound with insufficient evidence for definitive identification, but for which at least a tentative name and molecular formula could be proposed based on spectral data.

**Table 3:** Variance contribution rate and cumulative variance contribution rate of *Bidens pilosa* L. volatile oils.

Principal Component	Eigenvalue	Variance Contribution Rate	Cumulative Variance Contribution Rate (%)	Peak Loadings	Top Contributing Peaks (∣Loading∣ ≥ 0.5)
PC1	19.119	44.462	44.462	Peak 5: +0.92, Peak 8: −0.87, Peak 39: +0.81	5, 8, 39 (All∣loading∣ ≥ 0.8)
PC2	9.565	22.244	66.706	Peak 1: +0.78, Peak 34: −0.72	1, 34 (∣loading∣ ≥ 0.7)
PC3	4.731	11.003	77.709	Peak 20: +0.69, Peak 27: −0.64	20, 27 (∣loading∣ ≥ 0.6)
PC4	4.079	9.487	87.196	Peak 31: +0.58, Peak 38: −0.53	31, 38 (∣loading∣ ≥ 0.5)
PC5	2.111	4.910	92.106	Peak 2: +0.48, Peak 43: −0.42	None (all∣loading∣ < 0.5)
PC6	1.539	3.580	95.686	Peak 35: +0.41	None (all∣loading∣< 0.4)

**Table 4:** Principal component rotation component matrix analysis.

Compound	Component					
	1	2	3	4	5	6
P1	0.222	0.831	−0.211	−0.018	−0.073	−0.145
P2	0.231	0.443	−0.015	0.358	0.702	0.271
P3	−0.215	0.217	0.535	0.238	0.563	−0.352
P4	0.628	0.725	0.041	0.171	0.186	0.071
P5	0.985	0.113	0.021	−0.077	0	−0.057
P6	0.979	0.122	−0.004	0.111	0.04	−0.07
P7	0.657	0.271	0.16	0.53	0.403	0.12
P8	0.976	−0.162	−0.042	−0.077	−0.044	−0.091
P9	0.991	0.068	0.026	0.057	0.052	−0.028
P10	0.303	0.046	0.017	−0.185	0.923	−0.108
P11	0.885	−0.169	−0.189	−0.268	−0.099	−0.205
P12	0.156	−0.232	−0.177	−0.378	−0.678	0.215
P13	0.034	0.485	0.438	0.379	0.527	0.316
P14	0.284	0.904	0.138	0.114	0.233	0.062
P15	0.968	0.23	−0.034	−0.017	0.033	−0.06
P16	0.952	0.258	−0.07	−0.067	−0.022	0.097
P17	0.943	−0.229	0.196	0.051	0.015	0.059
P18	0.975	0.191	0.006	0.038	0.09	0.003
P19	0.848	0.136	−0.091	0.5	−0.001	0.026
P20	0.39	0.216	0.793	0.041	−0.1	0.253
P21	0.535	0.318	0.65	0.275	0.302	0.103
P22	0.964	−0.062	0.226	−0.031	0.09	−0.025
P23	0.952	0.249	0.005	−0.11	−0.001	0.052
P24	−0.27	0.539	−0.015	−0.22	−0.145	−0.107
P25	−0.087	0.001	0.982	0.07	0.116	−0.093
P26	−0.091	−0.027	0.984	−0.015	0.104	−0.099
P27	−0.027	0.232	0.963	0.054	0.09	−0.064
P28	0.064	0.921	0.272	0.052	0.214	0.067
P29	0.844	0.458	0.006	0.012	−0.024	0.268
P30	0.88	0.293	0.191	0.098	0.277	0.102
P31	0.165	0.023	0.535	0.568	0.549	0.241
P32	0.153	0.792	0.556	0.084	0.101	0.065
P33	0.278	0.791	0.241	−0.122	0.351	0.294
P34	0.463	0.776	0.175	0.059	0.348	0.094
P35	−0.067	0.577	−0.113	−0.09	0.043	0.768
P36	0.872	0.354	−0.026	0.099	0.203	0.16
P37	−0.018	−0.113	0.271	0.952	0.042	0.02
P38	0.039	0.494	−0.166	0.821	0.21	0.013
P39	0.648	0.617	0.212	0.252	0.276	0.099
P40	0.053	0.64	0.224	0.148	0.7	0.143
P41	−0.016	−0.094	0.054	0.985	0.117	−0.014
P42	−0.076	−0.12	−0.051	0.571	0.018	0.712
P43	−0.089	0.056	0.12	0.584	0.687	0.371

*Note*: The values in the table are loadings. These values represent the strength and direction of the correlation between the original variables (each compound) and the principal components. The larger the absolute value of a loading, the greater the contribution of that variable to the corresponding principal component.

**Table 5:** Principal component scores of volatile oil from *B. pilosa* L. from different origins.

Sample	Principal Component Score					
	1	2	3	4	5	6
S1	2.933	−0.463	−0.143	−0.361	−0.147	−0.271
S2	−0.043	−0.462	−0.384	2.943	0.174	−0.081
S3	−0.238	−0.361	−0.191	−0.251	−0.405	2.479
S4	−0.337	0.247	−0.481	−0.200	−0.952	−0.756
S5	0.170	2.910	−0.260	0.093	0.395	0.115
S6	−0.543	−0.577	−0.443	−0.429	−0.576	−0.377
S7	−0.531	−0.467	−0.456	−0.437	−0.287	−0.891
S8	−0.177	−0.015	2.991	0.090	−0.185	−0.203
S9	−0.351	−0.567	−0.156	−0.576	2.804	−0.218
S10	−0.246	−0.194	−0.182	−0.423	−0.218	1.137
S11	−0.637	−0.050	−0.295	−0.450	−0.603	−0.934

**Table 6:** Correlation between common peaks and antibacterial activity of 11 batches of *B. pilosa* L. volatile oil.

Common Peak	Grey Correlation Result			
	*E. coli*	*S. aureus*	*P. multocida*	*S. enterica*
P1	0.518	0.518	0.515	0.522
P2	0.67	0.682	0.697	0.713
P3	0.721	0.739	0.762	0.784
P4	0.496	0.497	0.501	0.496
P5	0.465	0.464	0.467	0.46
P6	0.453	0.452	0.454	0.447
P7	0.626	0.64	0.671	0.656
P8	0.866	0.805	0.737	0.721
P9	0.541	0.548	0.57	0.551
P10	0.533	0.536	0.542	0.54
P11	0.769	0.745	0.733	0.673
P12	0.976	0.957	0.832	0.871
P13	0.686	0.712	0.691	0.739
P14	0.476	0.475	0.475	0.472
P15	0.477	0.476	0.474	0.474
P16	0.591	0.602	0.63	0.612
P17	0.757	0.997	0.997	0.873
P18	0.545	0.552	0.57	0.555
P19	0.578	0.587	0.609	0.595
P20	0.443	0.534	0.527	0.596
P21	0.607	0.623	0.668	0.635
P22	0.745	0.769	0.814	0.815
P23	0.637	0.65	0.677	0.67
P24	0.637	0.65	0.677	0.669
P25	0.56	0.565	0.577	0.572
P26	0.691	0.713	0.76	0.742
P27	0.508	0.51	0.517	0.51
P28	0.46	0.46	0.463	0.455
P29	0.674	0.695	0.72	0.719
P30	0.604	0.617	0.652	0.629
P31	0.805	0.776	0.793	0.708
P32	0.48	0.481	0.486	0.478
P33	0.579	0.59	0.621	0.598
P34	0.497	0.5	0.51	0.498
P35	0.815	0.856	0.822	0.919
P36	0.642	0.652	0.661	0.677
P37	0.888	0.937	0.908	0.96
P38	0.483	0.484	0.489	0.481
P39	0.526	0.529	0.538	0.531
P40	0.543	0.549	0.562	0.552
P41	0.645	0.654	0.664	0.68
P42	0.547	0.51	0.455	0.473
P43	0.647	0.529	0.526	0.507

## Data Availability

All the data generated during this study are included in this published article and its supplementary information files, and the raw data used or analyzed during the current study are available from the corresponding author upon reasonable request.

## Supplementary Materials

The following supporting information can be downloaded at: https://www.mdpi.com/article/10.3390/plants15172665/s1, Figure S1: Overview of the main production areas for Chinese *Bidens pilosa* L. harvesting sites; Figure S2: Supercritical CO_2_ extraction of essential oil from *Bidens pilosa* L. harvested from different regions; Figure S3: Antibacterial profile of *B. pilosa* L. volatile oil from different batches; Table S1: Geographic and environmental information of the 11 *B. pilosa* sampling sites in China; Table S2: Index of Bidens L. in seeds; Table S3: The chemical components of the volatile oil in *Bidens pilosa* L.; Table S4: Antibacterial effects of *B. pilosa* volatile oils from different sources.

## Abbreviations

CFU, colony-forming unit; GC-MS, gas chromatography‒mass spectrometry; GRA, gray relational analysis; HCA, hierarchical cluster analysis; HPLC, high-performance liquid chromatography; KI, Kovats index; MIC, minimum inhibitory concentration; MS, mass spectrometry; NIST, National Institute of Standards and Technology; PCA, principal component analysis; PLS-DA, partial least squares-discriminant analysis; Q-marker, quality marker; RI, retention index; SCI, SFE-CO2, supercritical fluid extraction with carbon dioxide; TCM, traditional Chinese medicine; VIP, variable importance in projection.
