# Supplementary material for: Integrated GC-MS Fingerprinting and Chemometrics for Identifying Antimicrobial Quality Markers in Bidens pilosa L. Volatile Oils
Source: Plants (Basel). 2026 Aug 31;15(17):2665. doi: 10.3390/plants15172665 (PMC13567596; doi:10.3390/plants15172665)
Supplement: Supplementary file 1 [file plants-15-02665-s001.zip › plants-4469470-supplementary.pdf]

Figure legends

Figure S1 Overview of the main production areas for Chinese *Bidens pilosa* L. harvesting sites

Note: S1_Heibei Shijiazhuang、S2_Henan Nanyan、S3_Hubei Shenlongjia、S4_Anhui Bozhou、S5_Fujian Sanming、S6_Jiangxi Ganzhou、S7_Jiangxi zhangshu、S8_Guizhou Guiyang、S9_Shenxi Shangluo、S10_Guangdong Meizhou、S11_Guangxi Yulinlo

Figure S2 Supercritical CO_2 extraction of essential oil from *Bidens pilosa* L. harvested from different regions. S1_Heibei Shijiazhuang; S2_Henan Nanya; S3_Hubei Shenlongjia; S4_Anhui Bozhou; S5_Fujian Sanming; S6_Jiangxi Ganzhou; S7_Jiangxi zhangshu; S8_Guizhou Guiyang; S9_Shenxi Shangluo; S10_Guangdong Meizhou; S11_Guangxi Yulinlo

Figure S3 Antibacterial profile of *B. pilosa* L. volatile oil from different batches

Note: There are a total of 11 columns, each representing volatile oil from a different origin, a_Guangxi Yulin; b_Jiangxi Ganzhou; c_Fujian Sanming; d_Henan Nanyang; e_Shenxi Shangluo; f_Jiangxi Zhangshu; g_Guangdong Meizhou; h_Guizhou Guiyang; i_Anhui Bozhou; j_Hubei Shenglongjia; k_Hebei Shijiazhuang

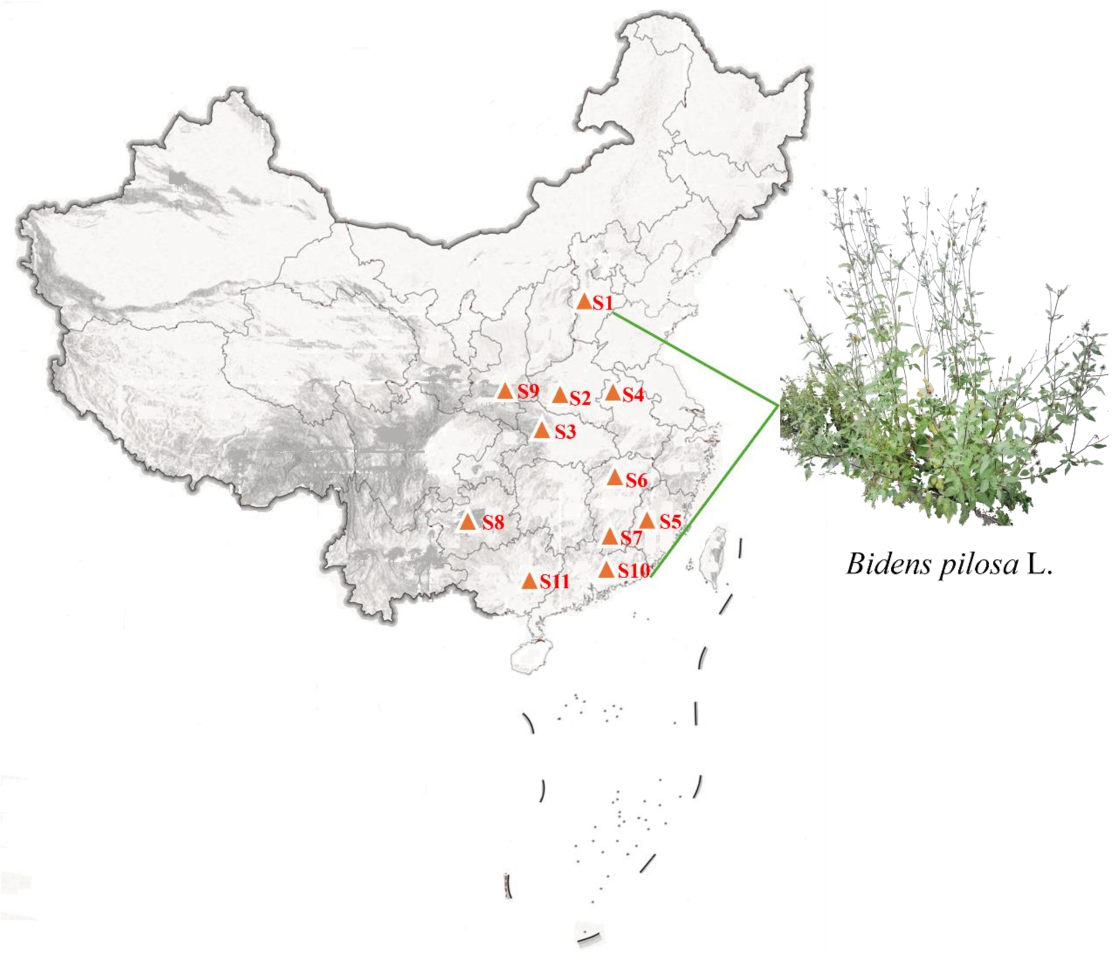

Figure S1

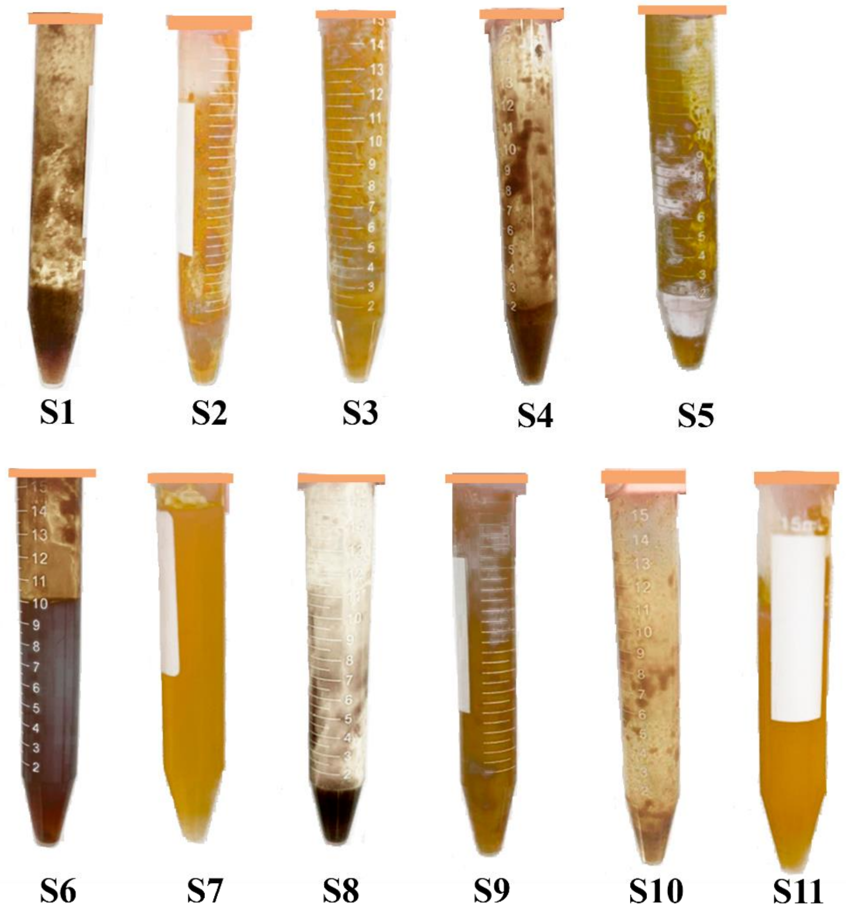

Figure S2

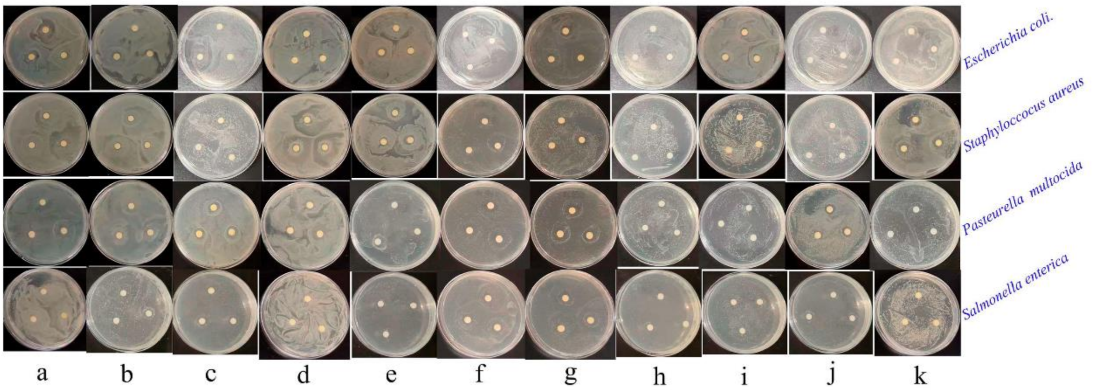

Escherichia coli.
Staphyloccocus aureus
Pasteurella multocida
Salmonella enterica
a
b
c
d
e
f
g
h
i
j
k

Figure S3

Table S1 Geographic and environmental information of the 11 *B. pilosa* sampling sites in China

Sample code	Location	Province	Climate type	Annual rainfall (mm)	GPS coordinates
S1	Shijiazhuang	Hebei	Temperate monsoon climate	401	38.0428° N, 114.5149° E
S2	Nanyang	Henan	Northern subtropical monsoon climate	804	32.9907° N, 112.5283° E
S3	Shennongjia	Hubei	Subtropical monsoon mountain climate	1000	31.7444° N, 110.6715° E
S4	Bozhou	Anhui	Warm temperate to northern subtropical monsoon climate	851	33.8466° N, 115.7793° E
S5	Sanming	Fujian	Subtropical monsoon climate	1650	26.2639° N, 117.6392° E
S6	Zhangshu	Jiangxi	Subtropical humid monsoon climate	1663	28.0559° N, 115.5462° E
S7	Ganzhou	Jiangxi	Subtropical humid monsoon climate	1605	25.8311° N, 114.9359° E
S8	Guiyang	Guizhou	Subtropical humid monsoon climate	1129	26.6470° N, 106.6302° E
S9	Shangluo	Shaanxi	Warm temperate monsoon climate	709	33.8683° N, 109.9244° E
S10	Meizhou	Guangdong	Subtropical monsoon climate	1483	24.2886° N, 116.1226° E
S11	Yulin	Guangxi	Subtropical monsoon climate	1650	22.6545° N, 110.1803° E

Note: GPS coordinates represent the central or representative locations of the sampling areas. Mean annual precipitation data were compiled from regional meteorological records and standard geographic references.

Table S2 Index of *Bidens* L. in seeds

#	Description of appearance/characteristics	Species*
1	The achene is oblong/elongated, four-angled or flattened four-angled, with a horseshoe-shaped hilum obliquely offset towards the dorsal side.	............②
1	Achene cuneate (wedge-shaped) or oblong-cuneate (oblong wedge-shaped), flattened, hilum central.	............⑤
2	Achene body 7-10 mm long, flattened four-angled, with upward-pointing, hair-like short spines on the ridges; apex with 3 (rarely 2) awns; achene surface with sparse or dense yellow tubercles.	............①
2	Achene body 7-12 mm or longer, surface without spines.	............③
3	Achene body 12-15 mm or longer, four-angled with 4 prominent longitudinal ridges, apex with 4 (rarely 3) awns; achene body sparsely covered with yellowish-brown tubercles.	............②
3	Achene body 15 mm or longer, slightly flattened except for the four angles at both ends, longitudinal ridges relatively flat, apex with 2 (rarely 3) awns.	............④
4	Achene body 12-15 mm long, with 3-4 fine ridges between the main ridges, achene body with upward-pointing short hairs; base horseshoe-shaped, hilum edge yellow with fine hairs, central depressed area dark brown.	............③
4	Achene body 20 mm long, with 1-2 fine ridges between the main ridges, achene body glabrous (hairless); base horseshoe-shaped, hilum edge yellow, smooth and glabrous, central depressed area yellow.	............④
5	Achene body slightly arched on the dorsal side, slightly depressed on the ventral side, apex slightly concave, mid-rib and edge ribs with upward-pointing hair-like spines.	............⑤

5	Achene body with indistinct dorsal and ventral sides, apex truncate (flat) or slightly convex, ridges with downward-pointing hair-like spines.	…………⑥
6	Achene body apex slightly convex, with pale yellow rounded humps, apex with 4 awns, all four ridges with barbs (retrorse spines).	…………⑥
6	Achene body apex truncate (flat), with 2 (rarely 3) awns, only the edge ridges with barbs (retrorse spines).	…………⑦
7	Achene body 5 mm long, obtriangular-cuneate (inverted triangular wedge-shaped), with pale yellow broad wings on the margins.	…………⑦
7	Achene body 10-12 mm long, oblong-cuneate (oblong wedge-shaped), margins with sharp ridges.	…………⑧

: ①_*Bidens pilosa L.; ②_*Bidens bipinnata* L.; ③_*Bidens parviflora* Willd.; ④_*Bidens biternata* (Lour.) Merr. et Sherff.; ⑤_*Bidens frondosa* L.; ⑥_*Bidens cernua* L.; ⑦_*Bidens maximowicziana* Oett; ⑧_*Bidens tripartite* L. refer to https://www.teagasc.ie/media/website/crops/horticulture/vegetables/Illustrated_Guide_to_Horticultural_Weeds_2020.pdf

Table S3 The chemical components of the volatile oil in *Bidens pilosa* L.

Serial Number	RT	Compounds	Molecular formula	CAS	KI (Calculated Kovats)	RI #(HP-5MS)	relative abundance (%)
1	9.1	cis-verbenol	$C_{10}H_{16}O$	473-67-6	1126	1133	0.01
2	9.2	verbenone	$C_{10}H_{14}O$	18309-32-5	1205	1203	0.02
3	10.2	3-ethyl-4-methyl-pyrrole-2,5-dione	$C_7H_9NO_2$	20189-42-8	1211	—	0.01
4	12.1	4-methyldodecane	$C_{13}H_{28}$	6117-97-1	1267	—	0.01
5	12.8	cis-cyclohexane-1,4-dimethanol diacetate	$C_{12}H_{20}O_4$	6308-18-5	1354	—	0.02
6	13.2	methyl laurate	$C_{13}H_{26}O_2$	111-82-0	1370	1365	25.53
7	13.3	β-bourbonene	$C_{15}H_{24}$	5208-59-3	1384	1386	0.01
8	13.9	caryophyllene	$C_{15}H_{24}$	87-44-5	1413	1419	0.05
8	14.5	α-humulene	$C_{15}H_{24}$	6753-98-6	1448	1453	0.03
9	14.8	β-ionone	$C_{13}H_{20}O$	79-77-6	1457	1469	0.01
10	14.9	(S,1Z,6*Z*)-8-isopropyl-5-methylene-1-methylcyclodeca-1,6-diene	$C_{15}H_{24}$	23986-74-5	1460	—	0.01
11	18.3	1-isopropyl-4,7-dimethyl-1,2,4a,5,6,8a-hexahydronaphthalene	$C_{15}H_{24}$	483-75-0	1476		0.01
12	15.2	γ-muurolene	$C_{15}H_{24}$	30021-74-0	1474	1481	22.05
13	15.4	β-bisabolene	$C_{15}H_{24}$	495-61-4	1480	1489	0.03
14	15.5	myristic acid	$C_{14}H_{28}O_2$	544-63-8	1510	1508	0.01
15	15.7	dihydroactinidiolide	$C_{11}H_{16}O_2$	17092-92-1	1531	1525	0.01
16	15.9	(±)-trans-nerolidol	$C_{15}H_{26}O$	40716-66-3	1565	1563	0.06
17	16.1	(-)-caryophyllene oxide	$C_{15}H_{24}O$	1139-30-6	1589	1561	0.08
18	16.2	mintoxide	$C_{15}H_{24}O$	88395-47-5	1591	1573	0.01
19	16.5	(*Z*)-3-heptadecene-5-yne	$C_{17}H_{30}$	74744-55-1	1595	—	0.02
20	16.6	salvial-4(14)-en-1-one	$C_{15}H_{24}O$	73809-82-2	1605	1599	0.01
21	16.7	fluorene	$C_{13}H_{10}$	86-73-7	1610	—	0.02

22	16.9	humulene epoxide II	$C_{15}H_{24}O$	19888-34-7	1614	1606	0.16
23	16.9	2,5-octadecadiynoic acid methyl ester	$C_{19}H_{30}O_2$	57156-91-9	1622	—	0.04
24	17.0	isophytol	$C_{20}H_{40}O$	505-32-8	1623	—	0.10
25	17.1	isospathulenol	$C_{15}H_{24}O$	88395-46-4	1625	—	0.03
26	17.2	β-longipinene	$C_{15}H_{24}$	41432-70-6	1635	1632	0.22
27	17.3	9h-fluorene	$C_{13}H_{10}$	86-73-7	1638	—	0.17
28	17.4	aromadendrene oxide II	$C_{15}H_{24}O$	85710-39-0	1658	1647	0.04
29	17.5	1-(2,4-dimethylphenyl)-2-methylpropan-1-one	$C_{12}H_{16}O$	23351-72-6	1662	—	0.20
30	17.6	octadec-9-yne	$C_{18}H_{34}$	35365-59-4	1730	—	0.14
31	19.4	11-hexadecyn-1-ol	$C_{16}H_{30}O$	65686-49-9	1758	—	0.09
32	19.5	1,4-cycloheptadiene,6-butyl-	$C_{11}H_{18}$	22735-58-6	1799	—	0.07
34	19.8	(*Z*)-14-methylhexadec-8-enal	$C_{17}H_{32}O$	60609-53-2	1825	—	0.05
35	19.9	neophytadiene	$C_{20}H_{38}$	504-96-1	1844	1840	0.11
36	20.0	phytone	$C_{18}H_{36}O$	502-69-2	1846	1838	0.04
37	20.2	dibutyl phthalate	$C_{16}H_{22}O_4$	84-74-2	1889	1967	0.24
38	20.5	n-pentadecanoic acid	$C_{15}H_{30}O_2$	1002-84-2	1912	—	0.01
39	21.5	ethyl pentadecanoate	$C_{17}H_{34}O_2$	41114-00-5	1960	—	0.34
40	21.6	(*E*,*E*,*E*,*E*,*E*,*E*)-(±)-2,6,10,15,19,23-hexamethyltetracosa-1,6,10,14,18,22-hexaen-3-ol	$C_{30}H_{50}O$	97232-74-1	1980	—	0.77
41	21.7	kauran-18-al, 17-(acetyloxy)-, (4β)-	$C_{22}H_{34}O_3$	41756-46-1	1995	—	0.06
42	21.9	palmitic acid	$C_{16}H_{32}O_2$	57-10-3	2012	—	0.03
43	22.0	stearic acid	$C_{18}H_{36}O_2$	57-11-4	2102	2188	11.33
44	22.2	phytol	$C_{20}H_{40}O$	150-86-7	2108	—	0.32
45	22.6	stearolic acid	$C_{18}H_{32}O_2$	506-24-1	2111	—	8.21
46	23.5	2-N-methylharmine	$C_{13}H_{12}N_2$	6519-18-2	2123	—	0.02
47	24.3	n-heneicosane	$C_{21}H_{44}$	629-94-7	2135	—	1.25

48	25.6	octacosane	$C_{28}H_{58}$	630-02-4	2146	—	2.33
49	25.9	4,8,12,16-tetramethylheptadecan-4-olide	$C_{21}H_{40}O_2$	96168-15-9	2150	—	0.01
50	26.6	tetracosanal	$C_{24}H_{48}O$	57866-08-7	2162	—	1.32
51	27.0	octadecanaldehyde	$C_{18}H_{36}O$	638-66-4	2170	—	0.02
52	27.5	nonadecane	$C_{19}H_{40}$	629-92-5	2320	—	0.71
53	28.5	behenic acid ethyl ester	$C_{24}H_{48}O_2$	5908-87-2	2482	—	0.22
54	29.2	α-tocospiro B	$C_{28}H_{48}O_4$	—	2530	—	0.33
55	32.4	stigmasteryl acetate	$C_{31}H_{50}O_2$	4651-48-3	2552	—	3.22
56	32.5	octacosanal	$C_{28}H_{56}O$	22725-64-0	2566	—	1.39
57	32.6	hentriacontane	$C_{31}H_{64}$	630-04-6	2580	—	0.22
58	32.8	vitamin E	$C_{29}H_{50}O_2$	1406-18-4	2586	—	0.74
59	33.2	dotriacontane	$C_{32}H_{66}$	544-85-4	2596	—	2.32
60	33.6	phytyl tetradecanoate	$C_{34}H_{66}O_2$	62172-52-5	2614	—	0.21
61	33.9	campasterol	$C_{28}H_{48}O$	474-62-4	2622	—	0.33
62	34.1	stigmasterol	$C_{29}H_{48}O$	83-48-7	2634	—	1.62
63	34.3	tritriacontane	$C_{33}H_{68}$	630-05-7	2648	—	1.59
64	34.6	β-sitosterol	$C_{29}H_{50}O$	83-46-5	2655	—	1.09
65	35.2	β-amyrin	$C_{30}H_{50}O$	559-70-6	2668	—	1.55
66	35.6	stigmast-8(14)-en-3β-ol	$C_{29}H_{50}O$	18069-99-3	2687	—	3.32
67	36.2	simiarenol	$C_{30}H_{50}O$	1615-94-7	2699	—	1.02
68	37.6	β-amyrin acetate	$C_{32}H_{52}O_2$	1616-93-9	2740	—	0.33
69	39.5	lupeol acetate	$C_{32}H_{52}O_2$	1617-68-1	2755	—	1.45
70	41.6	(*E*,*E*,*E*,*E*)-squalene	$C_{30}H_{50}$	111-02-4	2784	—	0.10

Table S4 Antibacterial effects of *B. pilosa* volatile oils from different sources

Sample	Inhibition zone diameter (mm)			
	E. coli	*S. aureus*	*P. multocida*	*S. enterica*
S1	8.62±0.53b	7.17±1.01d	7.77±0.30cd	6.32±0.01d
S2	7.22±1.17c	6.67±0.10de	6.18±0.08d	6.35±0.10d
S3	9.88±3.35a	8.20±0.41c	8.78±1.58c	9.38±0.62bc
S4	7.67±1.40c	6.15±0.02e	8.25±2.23c	6.33±0.01d
S5	6.90±0.79cd	7.10±0.95d	10.7±1.34b	9.83±0.81b
S6	8.08±1.15b	11.95±1.23a	12.5±1.46a	10.7±0.52b
S7	6.18±0.08d	7.03±0.23d	6.40±0.17d	8.63±0.74c
S8	6.75±0.46cd	9.10±0.92b	8.37±1.32c	10.17±1.25b
S9	8.27±0.10b	8.62±1.22c	9.90±1.09bc	8.82±0.53c
S10	10.42±0.68a	9.38±1.71b	12.93±0.35a	10.1±1.49b
S11	6.53±0.46cd	7.08±0.26d	6.12±0.04d	6.48±0.12d
blank control	—	—	—	—
positive control	10.29±0.31a	11.20±0.35a	10.17±0.31b	12.19±0.44a

Note: S1_Heibei Shijiazhuang, S2_Henan Nanyan, S3_Hubei Shenlongjia, S4_Anhui Bozhou, S5_Fujian Sanming, S6_Jiangxi Ganzhou, S7_Jiangxi zhangshu, S8_Guizhou Guiyang, S9_Shenxi Shangluo, S10_Guangdong Meizhou, S11_Guangxi Yulinlo. '-' indicates no visible inhibition zone; different letters in the same column (mean ± SD, n=3) indicate significant differences (P<0.05) between the tested volatile oil samples (S1-S11) and the positive control for the same bacterium. Blank control indicated 1% Tween-80. Positive control indicated 10 mg/mL ampicillin sodium
